# Carbamoyl-*N*-aryl-imine-urea: a new framework to obtain a putative leishmanicidal drug-candidate†

**DOI:** 10.1039/d0ra00287a

**Published:** 2020-03-26

**Authors:** Marina A. Alves, Aline C. de Queiroz, Anderson Brandão Leite, Felipe T. Martins, Antonio C. Doriguetto, Eliezer J. Barreiro, Magna S. Alexandre-Moreira, Lídia M. Lima

**Affiliations:** Instituto Nacional de Ciência e Tecnologia de Fármacos e Medicamentos (INCT-INOFAR), Laboratório de Avaliação e Síntese de Substâncias Bioativas (LASSBio, ®), Universidade Federal do Rio de Janeiro (UFRJ), CCS PO Box 68023, Cidade Universitária 21941-902 Rio de Janeiro RJ Brazil http://www.inct-inofar.ccs.ufrj.br http://www.lassbio.icb.ufrj.br; Programa de Pós-graduação em Química, Instituto de Química, UFRJ 21941-909 Rio de Janeiro RJ Brazil; Laboratório de Farmacologia e Imunologia, Instituto de Ciências Biológicas e da Saúde, Universidade Federal de Alagoas (UFAL) 57072-900 Maceió AL Brazil suzana.magna@gmail.com +55 82 3214 1528; Instituto de Química, Universidade Federal de Goiás (UFG) Campus Samambaia, CP 131 Goiânia GO 74001-970 Brazil

## Abstract

Leishmaniasis is a neglected parasitic disease, and current treatment includes limitations of toxicity, variable efficacy, high costs and inconvenient doses and treatment schedules. Therefore, new leishmanicidal drugs are still an unquestionable medical need. In this paper we described the design conception of a new framework, the carbamoyl-*N*-aryl-imine-urea, to obtain putative leishmanicidal drug-candidates. Compounds 9a–e and 10a–e were designed and synthesized and their leishmanicidal activity was studied in comparison to pentamidine, miltefosine and meglumine antimoniate. The conformational profile of the new carbamoyl-*N*-aryl-imine-urea framework was investigated by X-ray diffraction studies, using compound 9a as a model. The plasma stability of this putative peptide mimetic subunit was studied for compound 10e (LASSBio-1736). Among the congeneric series, LASSBio-1736 was identified as a new antileishmanial drug-candidate, displaying plasma stability, cytotoxicity against amastigote forms of *L. amazonensis* and *L. braziliensis*, and leishmanicidal activity in a cutaneous leishmaniasis murine model, without preliminary evidence of hepatic or renal toxicity.

## Abstract 2

LASSBio-1736 was identified as a new antileishmanial drug-candidate bearing an original framework and displaying plasma stability, cytotoxicity against amastigote forms of *Leishmania* species and leishmanicidal activity in a cutaneous leishmaniasis murine model.

## Graphical abstract

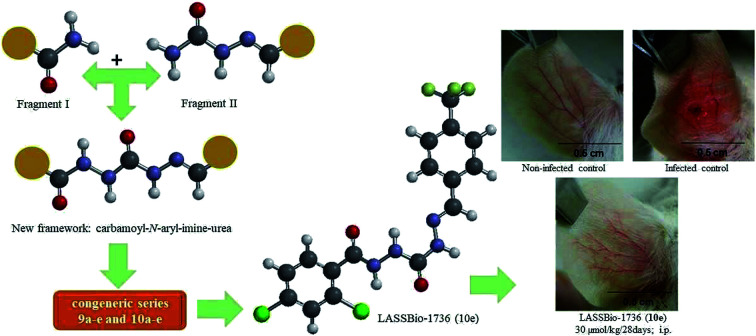

## Introduction

Leishmaniasis is a parasitic disease caused by about 20 *Leishmania* species and is transmitted to humans by more than 30 different species of phlebotomine sandflies. The parasitosis is clinically classified as (i) cutaneous leishmaniasis (CL), (ii) mucocutaneous leishmaniasis (ML), and (iii) visceral leishmaniasis (VL), the most severe form. Collectively they comprise one of the most prevalent neglected disease, with more than 2 million new cases occurring annually.^1^ Although leishmaniasis had been associated with risk factors such as socio-economic status, demographic area and human behaviors, an increase in its worldwide incidence has been attributed to migration, travel to endemic areas, deforestation, urbanization and organ transplant.^2^ The most common form of leishmaniasis is CL, which represents over 90% of cases distributed across three main endemic regions: (i) Afghanistan, Iran, Saudi Arabia and Syria; (ii) Algeria and Tunisia; and (iii) Brazil and Peru.^3^ It is mainly caused by *L. major* and *L. tropica* in the Old World, while *L. amazonensis*, *L. braziliensis* and *L. guyanensis* are responsible for the most cases of infection in the new world.

A promising strategy to treat parasitic infections is to inhibit parasite proteases. Also known as peptidases or peptide hydrolases, those enzymes are responsible by the catabolism of proteins and polypeptides through the cleavage of peptide bonds. Among peptidases subtypes, cysteinyl proteases (CPs) have been considered druggable targets for the design and development of new leishmanicidal drugs, since they play vital roles in the life cycle of *Leishmania* species.^4–6^

Numerous inhibitors of CPs have been described in the literature. They are designed considering the catalytic mechanism of cysteinyl proteases that predict the formation of a tetrahedral intermediate, generated from the nucleophilic addition of the cysteine residue to an electrophilic peptide carbonyl group.^7^ Two types of CPs inhibitors have been reported: peptide analogues and peptide mimetic derivatives. Their common feature is the presence of an electrophilic functionality that can be attacked by the catalytic cysteine thiolate in the active site. The peptide mimetic approach has been considered more advantageous, since it can overcome the undesirable drug-likeness properties associated with peptide structures, such as low solubility, poor bioavailability and metabolic instability.^8,9^

**Fig. 1:**
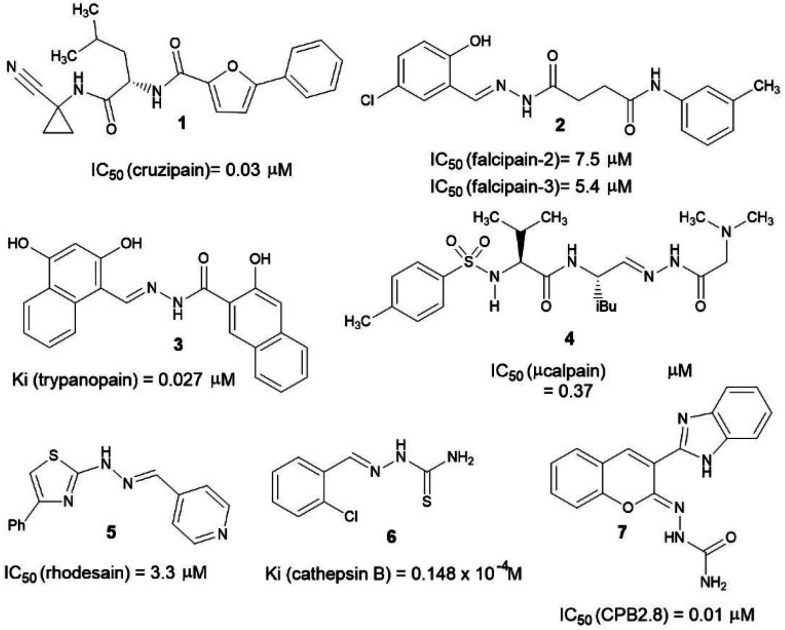
Examples of cysteine proteases inhibitors bearing a peptide mimetic framework.

Over the years, several chemotypes have been explored as a possible core to form transient covalent bond with sulfur atom of an essential cysteine residue from cysteinyl proteases, subsequently, inhibiting the catalytic function of those enzymes. Amide (1),^10^ acylhydrazone (2–4),^11–13^ hydrazone (5),^14^ thiosemicarbazone (6)^15^ and semicarbazone (7)^16^ are amongst the most prevalent chemotypes (Fig. 1).

In this paper we describe the design, synthesis, plasma stability and leishmanicidal activity of new compounds bearing a novel putative peptide mimetic framework: carbamoyl-*N*-aryl-imine-urea.

**Fig. 2:**
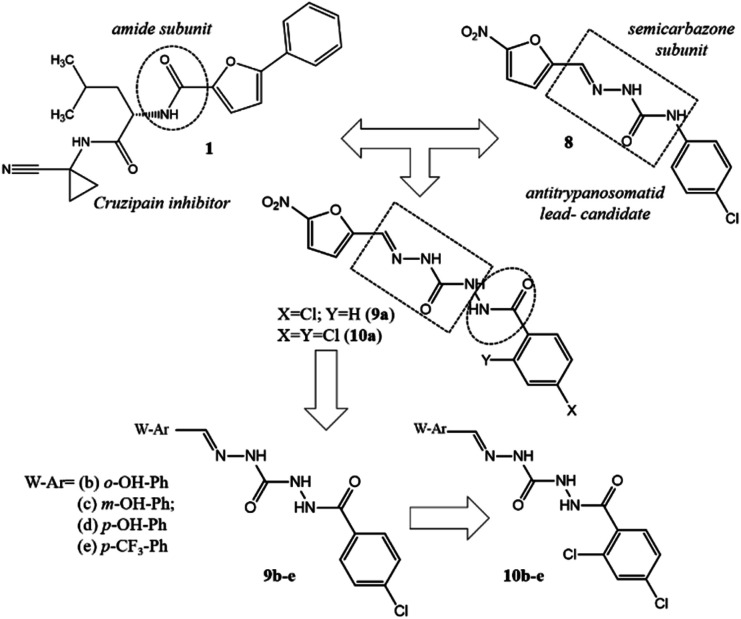
Design conception of compounds 9a–e and 10a–e bearing a new peptide mimetic framework – the carbamoyl-*N*-aryl-imine-urea.

Adopting the classical ligand-based drug design approach, the new carbamoyl-*N*-aryl-imine-ureas 9a–e and 10a–e were planned by molecular modifications at the prototype 8. The design conception considered the hybridization among the amide (RCONHR) and semicarbazone (RNHCONHN=CHR) fragments of the prototypes 1 and 8, respectively (Fig. 2). From this approach, compound 9a was designed holding a new framework as a linker to connect the aromatic rings, presented in the lead-compound 8. In view of the toxicophoric profile of 5-nitrofuranyl subunit,^17^ it was further replaced by phenyl ring. Substitution on the phenyl ring by electron donating and electron withdrawing groups allowing the conception of compounds 9b–e. To investigate the ortho-effect on the activity of compounds 9a–e, their analogues containing the chloro atom introduced in the position 2 of the phenyl ring linked to carbonyl subunit (10a–e) were also proposed (Fig. 2).

## Results and discussion

### Synthesis

**Fig. 3:**
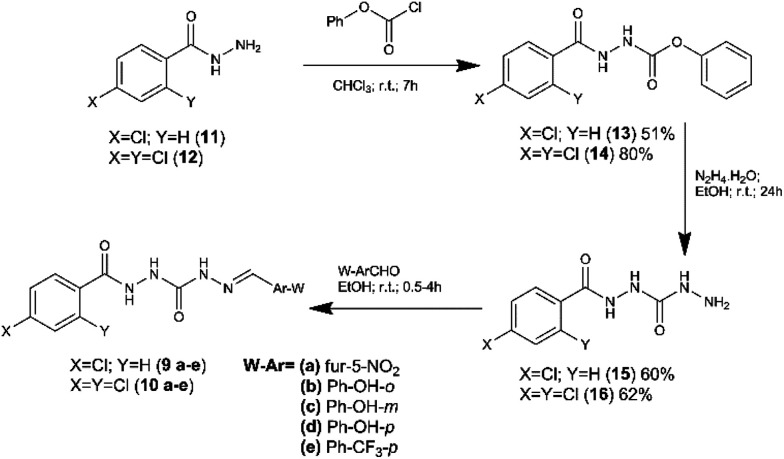
Synthetic route to obtain the target compounds 9a–e and 10a–e.

Compounds 9a–e and 10a–e were synthetized in three linear steps from the hydrazides 11 and 12, obtained commercially (Fig. 3). First, the phenyl carbamates (13 and 14) were prepared from compounds 11 and 12 by condensation reaction with phenyl chloroformate, using chloroform as solvent at room temperature.^18^ The key intermediates 15 and 16 were obtained in good yield from the carbamates 13 and 14 (respectively), exploring hydrazinolysis step in the presence of hydrazine hydrate and ethanol as solvent at room temperature.^19^ The synthesis of the target compounds 9a–e and 10a–e was concluded by condensation of the key-intermediates (15 and 16) with functionalized aldehydes, in the presence of ethanol at room temperature.^19^ Compounds 9a–e and 10a–e were obtained in overall yield ranging from 53 to 95%.

The chemical structure of compounds 9a–e and 10a–e was elucidated by ^1^H and ^13^C NMR, IR and mass spectrometry. The analysis of those spectra data has revealed that all compounds were synthetized as a single diastereoisomer (*Z* or *E*).

### Single crystal structure determination

To determine unequivocally the relative stereochemistry of the imine double bond (N=CH) and the molecular structure of the new peptide mimetic derivatives, X-ray diffraction studies have been proposed. However, considering the difficulty of getting compounds 9a–e and 10a–e in crystalline form, the X-ray crystallography assays have been performed only with derivative 9a, obtained as crystal solid.

**Fig. 4:**
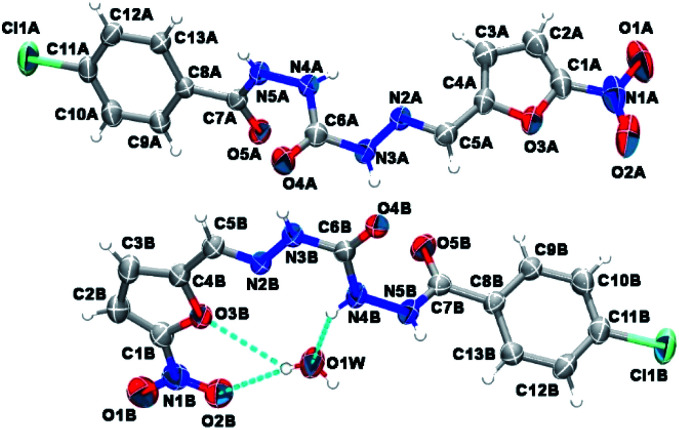
Asymmetric unit of compound 9a (LASSBio-1491) elucidated in this study. Non-hydrogen atoms are represented as 30% probability ellipsoids and randomly labeled, while hydrogens are shown as arbitrary radius spheres. Cyan dashed lines draw hydrogen bonds.

**Fig. 5:**
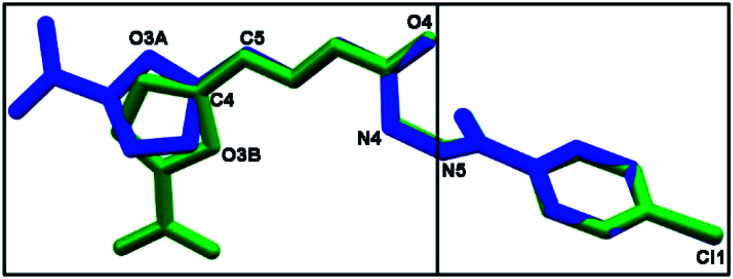
A molecular superposition of conformers A (blue) and B (green) of compound 9a (LASSBio-1491). Hydrogen atoms were omitted for clarity. Some key atoms were labeled to identify molecular fragments. The two molecular planes crossing through both conformers were split over two frames (left and right).

As depicted in Fig. 4, compound 9a (LASSBio-1491) has crystallized in the centrosymmetric monoclinic space group *P*2_1_/*c*. Crystal data and refinement results are listed in Table S1.† There were two molecules of compound 9a in the asymmetric unit (Fig. 4), which were labeled as A and B (atom labels ending with the corresponding capital letter). The occurrence of these two molecules features is a typical case of conformerism around only one rotatable single bond (Fig. 5). These two conformers differ only for a rotation of *ca.* 180° around the C4–C5 bond axis connecting the 5-nitrofuran ring to acylhydrazone group. The O3–C4–C5–N2 and C3–C4–C5–N2 torsions differ for 177.7 (10)° and 178.0 (13)° between the two conformers (O3–C4–C5–N2 = 175.8 (4)° in A and −1.9 (6)° in B; C3–C4–C5–N2 = −2.3 (8)° in A and 175.7 (5)° in B). This rotation changes the relative orientation of the heterocyclic O3 furan and the O4 carbonyl oxygens in the conformers. While these oxygens are on the same side of the open chain encompassing them in conformer A, they are on opposite sides in conformer B. Except for this conformational difference, the remaining backbones of conformers A and B is similar [root mean square deviation (r.m.s.d.) of 0.156 Å for all overlaid corresponding non-hydrogen atoms from both conformers, excluding nitrofuran atoms O1–O3, N1, and C1–C3; see Fig. 5]. These equivalent backbones can be described by two molecular planes which are almost bent perpendicularly. This twist is gotten on the N4–N5 hydrazine bond (C6–N4–N5–C7 = −70.7 (5)° in A and −82.7 (5)° in B), which gives rise to one plane adjusted from N4 to nitro group (r.m.s.d. of the fourteen fitted non-hydrogen atoms is 0.116 Å in A and 0.0245 Å in B) and another one from N5 to Cl1 (r.m.s.d. of the ten fitted non-hydrogen atoms is 0.145 Å in A and 0.164 Å in B). These two molecular planes are bent by 69.88 (7)° and 78.32 (7)° in molecules A and B, respectively (Fig. 5). A slight twist is also found in both conformers around the C7–C8 bond axis connecting the 4-chlorobenzene ring to carbonyl moiety (O5–C7–C8–C9 = 16.0 (6)° in A and 17.1 (6)° in B).

Besides the two conformers, there is also one water molecule in the asymmetric unit of compound 9a, revealing that this compound has crystallized as a hemihydrate. Water molecule selectively interacts only with conformer B, being even responsible for the conformational change found between the two conformers. The whole water molecules lie onto the molecular plane adjusted from N4 to nitro group of molecule B, where it acts as a hydrogen bonding acceptor from N4B–H4B and as a bifurcated hydrogen bonding donor to the oxygens O2B (heterocyclic furan oxygen) and O3B (from nitro group) (Fig. 4). These last contacts enforce the rotation about C4–C5, introducing conformerism in the crystal structure of the studied compound.

### Biological assays

#### 1 Cytotoxicity against J774.A1 macrophages

Initially the cytotoxic profile of compounds 9a–e and 10a–e against J774.A1 cell lines (*i.e.*, BALB/c macrophage cell lines) was investigated. Therefore, these cells were exposed to different concentrations (0.1–100 μM) of compounds 9a–e and 10a–e and the cytotoxic effect was measured after 48 h of drug incubation, by using MTT-based assay. The cytotoxicity was expressed as IC_50_ values (that means the concentration of a drug that kills half of the tested cells in culture). As depicted in Table 1, most compounds have shown low cytotoxic activity with IC_50_ > 100 μM. Compounds 9c, 9d, and 10b have displayed IC_50_ values of 85.2 μM, 86.2 μM, and 78.1 μM, respectively.

**Table 1:** Cytotoxicity of carbamoyl-*N*-aryl-imine-urea derivatives against macrophages (J774.A1 cell lines) measured by MTT assay

Compound	IC_50_^a^
Miltefosine	>100 μM
Pentamidine	35.5 ± 3.4 μM
LASSBio-1491 (9a)	>100 μM
LASSBio-1704 (9b)	>100 μM
LASSBio-1709 (9c)	85.2 ± 2.3 μM
LASSBio-1706 (9d)	86.2 ± 8.7 μM
LASSBio-1737 (9e)	>100 μM
LASSBio-1703 (10a)	>100 μM
LASSBio-1705 (10b)	78.1 ± 4.5 μM
LASSBio-1708 (10c)	>100 μM
LASSBio-1707 (10d)	>100 μM
LASSBio-1736 (10e)	>100 μM

a Concentration required to give 50% death of cells (IC_50_) was calculated by linear regression analysis from the culture growth constant (*K*_c_) values at employed concentrations (100, 10, 1 and 0.1 μM).^23^ This constant corresponds to the slope resulting from plotting the log of the growth measurement *versus* time for each drug concentration.

#### 2 Leishmanicidal activity against amastigote forms of *L. amazonensis* and *L. Braziliensis*

**Fig. 6:**
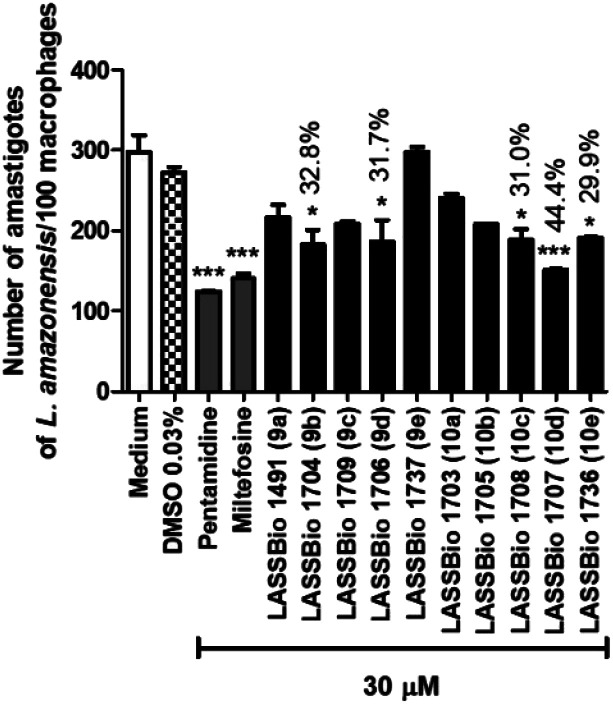
Effect of pentamidine and carbamoyl-*N*-aryl-imine-urea derivatives (9a–e and 10a–e) against intracellular amastigote forms of *L. amazonensis* at concentration of 30 μM.

Considering that *L. amazonensis* is one of the causative species of cutaneous leishmaniasis in Brazil,^20^ we have investigated the leishmanicidal effect of compounds 9a–e and 10a–e against *L. amazonensis* amastigotes forms. Initially, all compounds were evaluated at 30 μM. At this screening concentration, compounds 9b (LASSBio-1704), 9d (LASSBio-1706), 10c (LASSBio-1708), 10d (LASSBio-1707) and 10e (LASSBio-1736) decreased the number of intracellular amastigotes (Fig. 6). Then, they were selected to determine their IC_50_ value for intracellular amastigote forms of *L. amazonensis*. As demonstrated in Table 2, 9b and 10e were the most active amongst the evaluated compounds, showing IC_50_ values of 85.3 ± 5.0 μM and 84.0 ± 0.3 μM, respectively. These data reveal equipotency between them. However, they were about 2.5 times and 4.0 times less potent than the standards pentamidine (IC_50_ = 32.8 ± 4.6 μM) and miltefosine (IC_50_ = 22.0 ± 1.8 μM), respectively. All compounds displayed similar maximum effect (Table 2).

**Table 2:** *In vitro* leishmanicidal activity against intracellular amastigote forms of *L. amazonensis* and *L. braziliensis*

Compound	*L. amazonensis*		*L. braziliensis*	
	IC_50_^a^ (μM)	Maximum effect^b^ (% ± S.E.M.)	IC_50_^a^ (μM)	Maximum effect^b^ (% ± S.E.M.)
Miltefosine	22.0 ± 1.8	58.1 ± 4.9**	32.1 ± 1.1 μM	84.0 ± 1.5**
Pentamidine	32.8 ± 4.6	59.1 ± 5.6**	78.4 ± 4.7 μM	61.7 ± 2.2**
LASSBio-1704 (9b)	85.3 ± 5.0	55.6 ± 1.4**	>100 μM	48.1 ± 4.5**
LASSBio-1706 (9d)	>100	NA	3.6 ± 0.6 μM	70.4 ± 1.9**
LASSBio-1708 (10c)	>100	31.0 ± 1.7**	>100 μM	NA
LASSBio-1707 (10d)	>100	44.4 ± 0.2**	>100 μM	NA
LASSBio-1736 (10e)	84.0 ± 0.3	57.6 ± 0.2**	5.3 ± 2.6 μM	57.2 ± 5.2**

a IC_50_ is the concentration required to give 50% death of parasites, calculated by linear regression analysis from the *K*_c_ values at employed concentrations (100, 10, 1, 10^−1^, 10^−2^ and 10^−3^ μM).^24^ This constant corresponds to the slope resulting from plotting the log of the growth measurement *versus* time for each drug concentration.

b Maximum effect (ME) is expressed as mean ± standard error of maximum toxicity average of triplicates of a representative experiment. The values of maximum effect were considered significant when *p < 0.05, **p < 0.01 compared to the 0.1% DMSO group.

Further, the leishmanicidal activity against intracellular amastigotes forms of *L. braziliensis* were also investigated (Table 2). The carbamoyl-*N*-arylimine-ureas 9d (LASSBio-1706) and 10e (LASSBio-1736) showed anti-amastigote activity with IC_50_ values of 3.6 μM and 5.3 μM, respectively. These results revealed the higher potency of 9d and 10e in comparison with the standard drugs miltefosine (IC_50_ = 32.1 μM) and pentamidine (IC_50_ = 78.4 μM).

As shown in Table 2, significant variations in activity of carbamoyl-*N*-arylimine-ureas were observed against the intracellular amastigote of *L. amazonensis* and of *L. braziliensis*, which can be owing the variances among both *Leishmania* spp. or due the differences in the mechanism of action of those compounds when compared to the standards pentamidine and miltefosine.

#### 3 Plasma stability

**Fig. 7:**
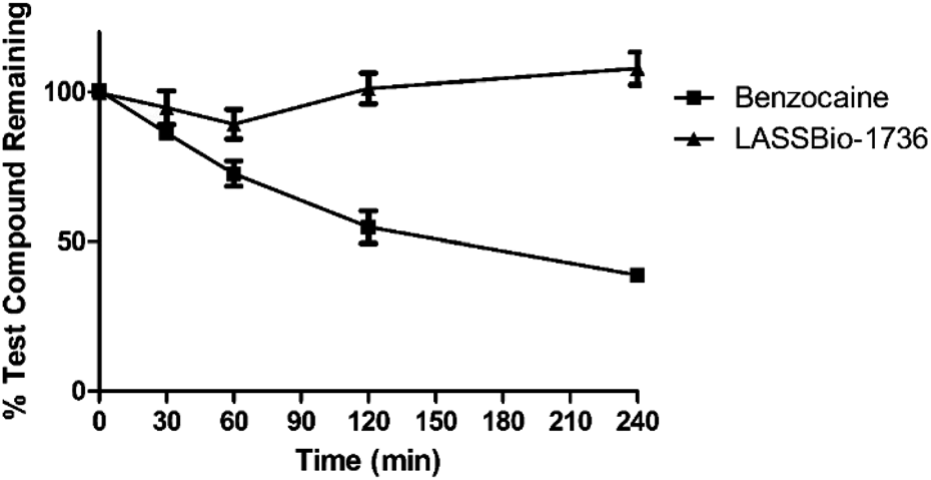
Plasma stability of 10e (LASSBio-1736) ▲ and standard benzocaine ■. Data were obtained through the compound recovery percentage analyzed by UPLC-MS/MS.

To investigate the potential plasma instability of the carbamoyl-*N*-arylimine-urea subunit, designed as a new peptide mimetic framework, compound 10e (LASSBio-1736) was selected to investigate its plasma stability,^21,22^ previously to the evaluation of its *in vivo* leishmanicidal effect. Quantitation of 10e (LASSBio-1736), benzocaine standard (positive control; it means a substrate drug for plasma hydrolysis) and ketoconazole (internal standard) was determined by UPLC-MS/MS, after incubation of the compounds at 0, 30, 60, 120 and 240 min with rat plasma, and measuring the peak areas in three different experiments. As demonstrated in Fig. 7, in comparison with the positive control benzocaine, which was partially recovered (38%) after 4 hours of incubation with rat plasma, 10e (LASSBio-1736) was completely recovered, exhibiting a great plasma stability. It was also stable to hydrolysis at PBS (pH 7.4) (Fig. S38†).

#### 4 *In vivo* leishmanicidal activity in a murine model of cutaneous leishmaniases

Considering the low cytotoxic activity of compound 10e (LASSBio-1736) against J774.A1 cell line (Table 1), its leishmanicidal activity against amastigote forms of *L. amazonensis* and *L. braziliensis* (Table 2) and its great plasma stability (Fig. 7), it was selected to be studied in a murine model of cutaneous leishmaniasis induced by *L. amazonensis*.^25^

**Fig. 8:**
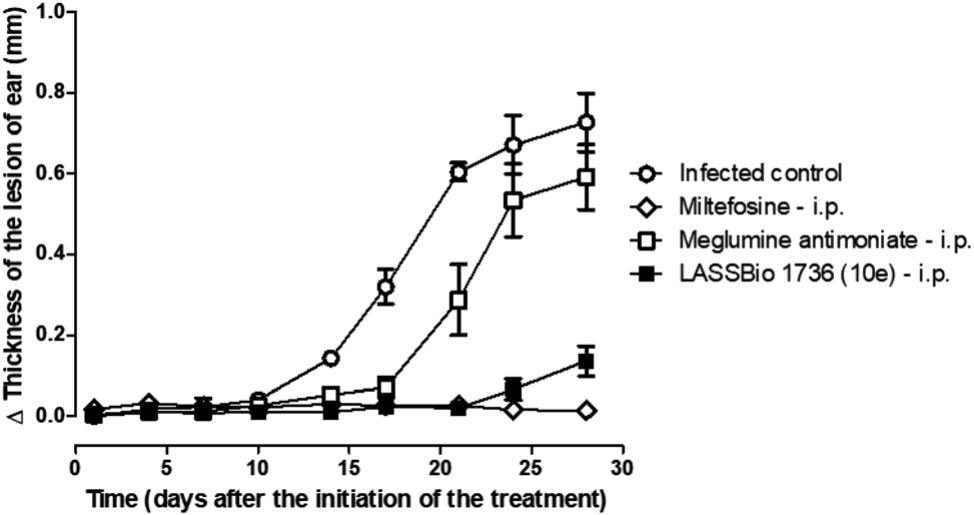
*In vivo* efficacy of meglumine antimoniate, miltefosine and 10e (LASSBio-1736) treatments (30 μmol per kg per day × 28 days) in BALB/c mice infected with *L. amazonensis*. Lesion sizes were monitored weekly. Values are the mean of lesion sizes in five mice in each group and bars represent the standard error of the mean.

**Fig. 9:**
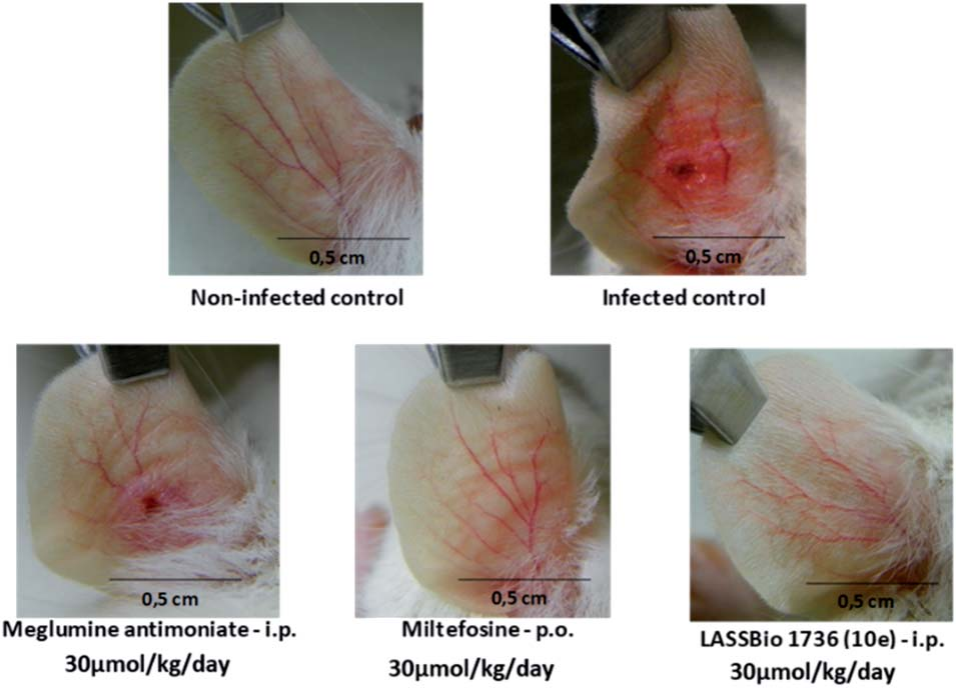
Images of lesions after of treatment of BALB/c mice infected with *L. amazonensis* untreated and treated with meglumine antimoniate, miltefosine and 10e (LASSBio-1736) at dose 30 μmol per kg per day × 28 days. Photographs were taken on the first day after end of treatment. In the control groups, infected control, and meglumine antimoniate, the lesions showed an intense swelling and ulcerated by after treatment cessation. In groups treated with miltefosine and 10e (LASSBio-1736), the photographs revealed a complete healing of the nodules and ulcers.

So, BALB/c mice infected with *L. amazonensis* were treated during 28 days with compound 10e (30 μmol per kg per day, i.p.) or with the standards miltefosine (30 μmol per kg per day, p.o.) and meglumine antimoniate (30 μmol per kg per day, i.p.). As demonstrated in Fig. 8 and 9, the carbamoyl-*N*-arylimine-urea 10e (LASSBio-1736) has reduced the lesion sizes of infected ear like miltefosine, and both have shown better efficacy than meglumine antimoniate.

**Fig. 10:**
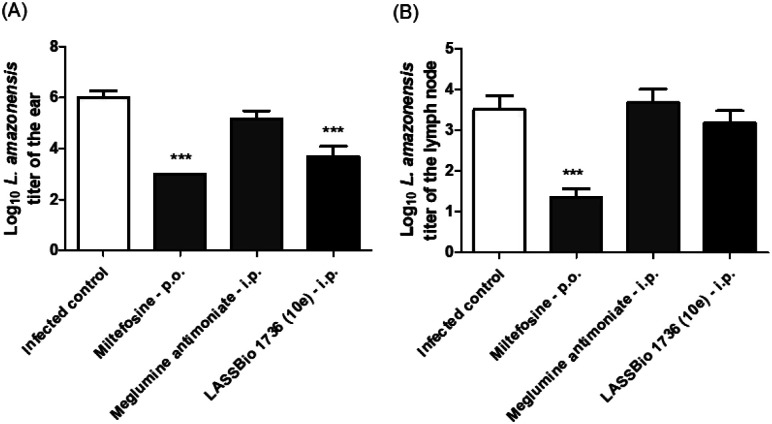
Parasite burden throughout the course of miltefosine (30 μmol per kg per day × 28 days, p.o.), meglumine antimoniate and LASSBio-1736 (10e) (30 μmol per kg per day × 28 days, i.p.) treatments in BALB/c mice infected with *L. amazonensis*. (A) log_10_ of parasites loads of the infected ear. (B) log_10_ of parasites loads of the draining lymph node. The parasite loads of infected ears and draining lymph nodes were determined using a quantitative limiting-dilution assay. Values are the mean of parasites loads in five mice in each group and bars represent the standard error of the mean. ***P < 0.001 *vs.* control.

Regarding the parasite burden, 10e has decreased parasite load in infected ear as miltefosine, but it has not reduced the *L. amazonensis* titer in draining lymph node (Fig. 10).

**Fig. 11:**
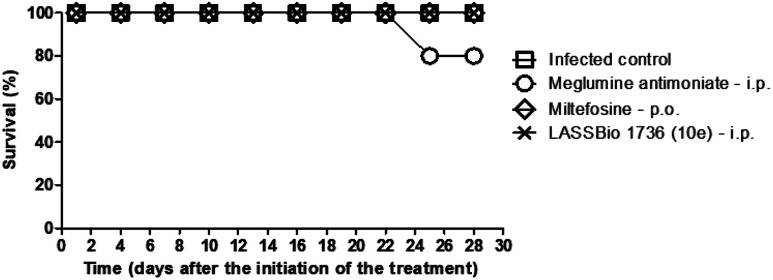
Survival of BALB/c mice infected with *L. amazonensis* untreated and treated with meglumine antimoniate, miltefosine, and LASSBio-1736 (10e) at dose 30 μmol per kg per day × 28 days. Data are from representative experiments, *n* = 5 mice per group. Treatment with meglumine antimoniate induced 20% of mortality in the group and other treatments presented survival rate of 100%.

**Fig. 12:**
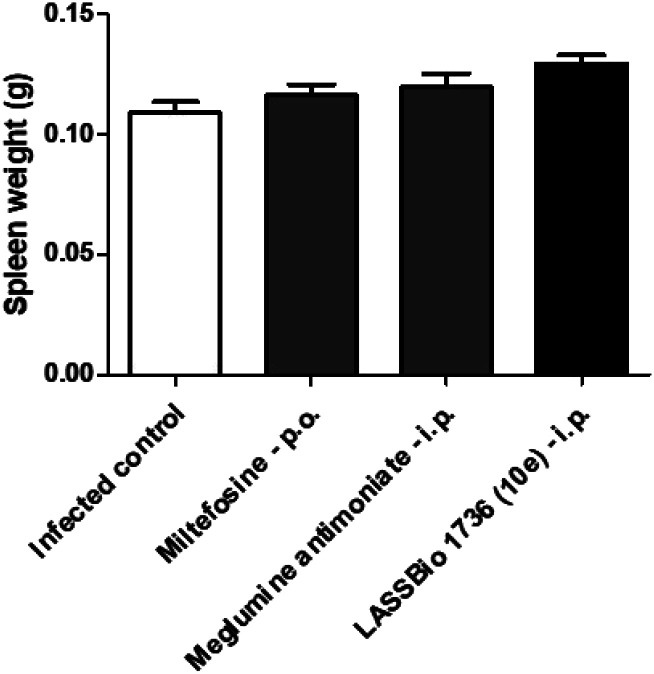
*In vivo* effect of miltefosine (30 μmol per kg per day × 28 days, p.o.), meglumine antimoniate and LASSBio-1736 (10e) (30 μmol per kg per day × 28 days, i.p.) in spleen weight of BALB/c mice infected with *L. amazonensis*. Spleen weight was verified in last day of treatment. Values are the mean of the spleen weight in five mice in each group and bars represent the standard error of the mean. *P < 0.05 *vs.* control.

**Fig. 13:**
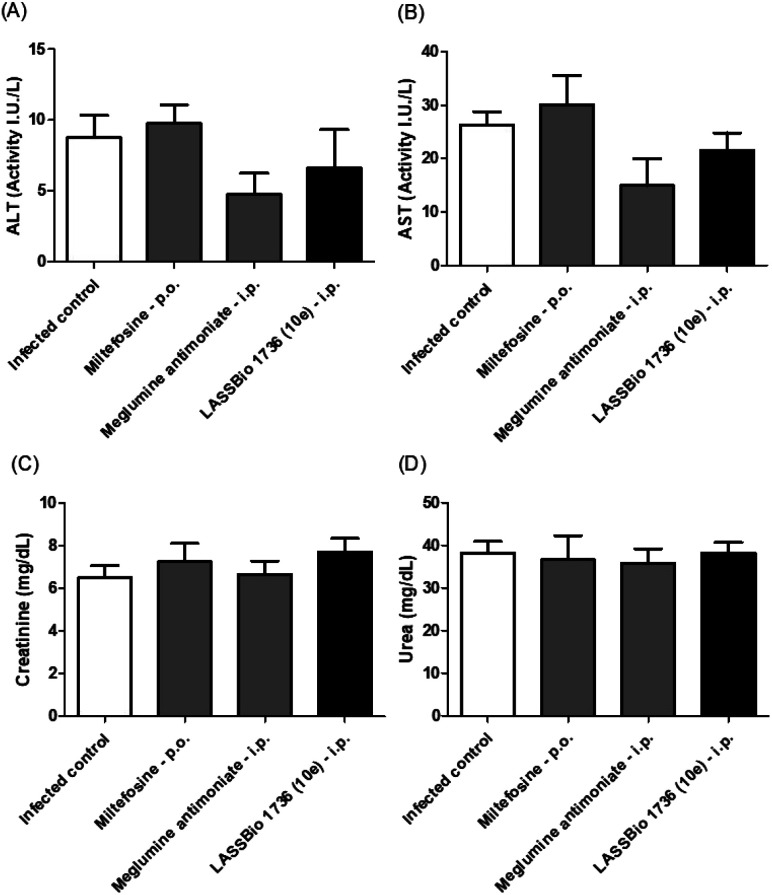
*In vivo* effect of miltefosine (30 μmol per kg per day × 28 days, p.o.), meglumine antimoniate and LASSBio-1736 (10e) (30 μmol per kg per day × 28 days, i.p.) treatments on serum ALT (A), AST (B), creatinine (C) and urea (D) levels of BALB/c mice infected with *L. amazonensis*. Values are the mean of the plasma levels of biochemical parameters in five mice in each group and bars represent the standard error of the mean. *P < 0.05, **P < 0.01 *vs.* control.

The survival of BALB/c mice infected with *L. amazonensis* untreated and treated during 28 days with the daily dose of 30 μmol kg^−1^ with meglumine antimoniate, miltefosine and 10e (LASSBio-1736) was analyzed. Animal mortality was not observed for the groups treated with 10e and miltefosine, while 20% of death was identified from the 25^th^ day of treatment with meglumine antimoniate (Fig. 11). After the end of treatment, the animal's toxicity was investigated looking for any alterations on spleen weight and on alanine aminotransferase (ALT), aspartate aminotransferase (AST), creatinine (CREA) and urea serum levels. As shown in Fig. 12 and 13 compound 10e (LASSBio-1736) did not significantly interfere with serum levels of these biochemical markers, suggesting no hepato- and nephrotoxic activities. Moreover, like the standards drugs, 10e (LASSBio-1736) did not alter animal's spleen weight.

## Experimental section

### Chemistry

Reagents and solvents were purchased from commercial suppliers. The reactions were monitored by thin layer chromatography, which was performed on aluminum sheets pre-coated with silica gel 60 (HF-254, Merck) to a thickness of 0.25 mm. The chromatograms were viewed under ultraviolet light (254–265 nm). ^1^H NMR and ^13^C NMR spectra were determined in deuterated dimethyl sulfoxide using a Bruker DPX-200 at 200 MHz, Varian Mercury-300 (300 MHz), Varian MR-400 (400 MHz). Signal multiplicities are represented by: s (singlet), d (doublet), t (triplet), q (quadruplet), m (multiplet) and br (broad signal). Infrared (IR) spectra were obtained with a FTLA 2000-100 spectrophotometer using potassium bromide plates. Melting points of final products were determined with a Quimis 340 apparatus and are uncorrected. The purity of compounds was determined by HPLC (>95%) using the Shimadzu LC20AD apparatus, a Kromasil 100-5C18 (4.6 mm × 250 mm) column and the SPD-M20A detector (Diode Array) at 220–310 nm for quantification of analyte in a 1 mL min^−1^ constant flux. The injector was programmed to inject a volume of 20 μL. The mobile phases used were: CH_3_CN : H_2_O 1 : 1; 4 : 1; 6 : 4 and 7 : 3. The results of elemental analysis were obtained FlashEA 1112 Series instrument (Thermo Scientific, Waltham, USA) from samples previously dried under vacuum. The HRMS analysis was performed using a QExactive™ Hybrid Quadrupole Orbitrap Mass Spectrometer (Thermo Fisher Scientific, Waltham, USA) using electrospray ionization (ESI). Standard working solutions of compounds 9a–e and 10a–e (1 μg mL^−1^) were prepared with methanol/water 7 : 3 and fortified with 0.1% formic acid and 5 mM NH_4_COOH (ammonium formate), and the solutions were used by direct infusion.

#### 5 General procedure for the preparation of carbamate intermediates-compounds 13 and 14 (adapted from ref. 18)

Phenyl chloroformate (1.1 mL, 8 mmol, 1.2 eq.) was dissolved in 20 mL of chloroform. The solution was stirred at room temperature and added slowly a suspension of 1.5 g (7.3 mmol) of compounds 11 (4-chlorobenzhydrazide) or 12 (2,4-dichlorobenzhydrazide) in 50 mL of chloroform. After 7 hours at room temperature, complete consumption of the starting materials was observed by TLC (eluent: dichloromethane : methanol 5%). Then, 10 mL of hexane was added to the reaction, keeping it under stirring for 10 minutes. Following, it was vacuum filtered and washed with hexane to give the product 13 or 14.

##### 5.1. Preparation of the 4-chloro-phenylcarbamoyl-*o*-phenylcarbamate (compound 13; LASSBio-1702)

Compound 13 was obtained in 70% of yield, as a white solid with mp = 182–185 °C. IR (KBr) (cm^−1^): 3277 (*ν*NH); 1730 (*ν*C=O); 1661 (*ν*C=O); 1010 (*δ* Ar-Cl); ^1^H-NMR (200 MHz, DMSO-d_6_) *δ* (ppm): 10.62 (s, 1H, Ar-CONH); 9.92 (s, 1H, NHCO); 7.90 (d, 2H, H2, H6, *J* = 10 Hz); 7.60 (d, 2H, H3, H5, *J* = 10 Hz); 7.44 (d, 2H, H3′, H5′, *J* = 8 Hz); 7.26 (t, 1H, H4, *J* = 6 Hz); 7.18 (d, 2H, H2′, H6′, *J* = 8 Hz); 97% purity in HPLC (R.T. = 3.6 min, CH_3_CN : H_2_O (6 : 1)); HRMS calculated for C_14_H_11_ClN_2_O_3_: [M + H]^+^ = 291.05309, found: *m*/*z* 291.05241.

##### 5.2. Preparation of 2,4-dichloro-phenylcarbamoyl-*o*-phenylcarbamate (compound 14; LASSBio-1492)

Compound 14 was obtained in 80% of yield, as a white solid with mp = 180–184 °C; IR (KBr) (cm^−1^): 3247 (*ν*NH); 1741 (*ν*C=O); 1661 (*ν*C=O); 1033 (*ν*Ar-Cl); ^1^H-NMR (200 MHz, DMSO-d_6_) *δ* (ppm): 10.49 (s, 1H, ArCONH); 10.05 (s, 1H, NHNHCO), 7.74 (d, 1H, H5, *J* = 2 Hz); 7.58–7.14 (m, 7H, H3, H6, H2′, H3′, H4′, H5′, H6′); ^13^C-NMR (50 MHz. DMSO-d_6_) *δ* (ppm): 163.1 (ArCONH), 154.2 (NHNHCO); 150.6 (C1′); 135.4 (C4), 133.1 (C1); 131.7 (C2); 130.5 (C4′); 129.5 (C3′, C5′); 129.4 (C2′, C6′); 127.4 (C3); 125.4 (C5); 121.5 (C6); 98% purity in HPLC (R.T. = 4.25 min, CH_3_CN : H_2_O (7 : 1)); HRMS calculated for C_14_H_10_Cl_2_N_2_O_3_: [M + H]^+^ = 325.01412, found: *m*/*z* 325.01385.

#### 6 General procedure for the preparation of urea intermediates 15 and 16 (adapted from ref. 19)

Starting from 0.3 g (1 mmol) of intermediate 13 or 14, was added 7 mL of absolute ethanol and 1.20 mL of hydrazine hydrate 80% (0.03 mol, 30 eq.). The resulting mixture was stirred at room temperature and after 32 hours was observed the end of the reaction by TLC (eluent: dichloromethane : methanol 5%). The reaction volume was reduced in vacuum system and after addition of ice occurred precipitation of the product, which was filtered under vacuum.

##### 6.1. Preparation of intermediate 4-chloro-phenylcarbamoyl-*N*-amine-urea (compound 15; LASSBio-1710)

Yield: 64%, white solid, mp 187–190 °C; IR (KBr) (cm^−1^): 3328 (*ν*NH); 1676 (*ν*C=O); 1597 (*ν*C=O); 1013 (*δ* Ar-Cl); ^1^H-NMR (200 MHz, DMSO-d_6_) *δ* (ppm): 10.24 (s, 1H, Ar-CONH); 8.17 (s, 1H, NHCONH); 7.89 (d, H2, H6, *J* = 8); 7.63 (s, 1H, NHCONH); 7.55 (d, 2H, H3, H5, *J* = 8); 4.17 (s, 2H, NH_2_); ^13^C-NMR (50 MHz. DMSO-d_6_) *δ* (ppm): 165.7 (Ar-CO); 160.3 (NHCONH); 136.9 (C4); 132.0 (C1); 129.9 (C2, C6); 128.9 (C3, C5); 97% purity in HPLC (R.T. = 3.6 min, CH_3_CN : H_2_O (6 : 1)); HRMS calculated for C_8_H_9_ClN_4_O_2_: [M + H]^+^ = 229.04867, found: *m*/*z* 229.04852.

##### 6.2. Preparation of intermediate 2,4-dichloro-phenylcarbamoyl-*N*-amine-urea (compound 16; LASSBio-1493)

Yield: 62%, white solid, mp 190–194 °C; IR (KBr) (cm^−1^): 3297 (*ν*NH); 1698, (*ν*C=O); 1651 (*ν*C=O); 1041 (*ν*C–Cl); ^1^H-NMR (200 MHz, DMSO-d_6_) *δ* (ppm): 10.17 (s, 1H, Ar-CONH); 8.24 (s, 1H, NHCONH); 7.70 (d, 1H, H5, *J* = 2 Hz); 7.60–7.50 (m, 3H, H3, H6, NHCONH); 4.15 (s, 2H, NH_2_); ^13^C-NMR (50 MHz. DMSO-d_6_) *δ* (ppm): 165.0 (Ar-CO); 159.3 (NHCONH); 135.0 (C4); 133.5 (C1); 131.7 (C2); 130.8 (C3); 128.3 (C5); 127.2 (C6); 97% purity in HPLC (R.T. = 3.6 min, CH_3_CN : H_2_O (6 : 1)); HRMS calculated for C_8_H_8_Cl_2_N_4_O_2_: [M + H]^+^ = 263.00970, found: *m*/*z* 263.00932.

#### 7 General procedure for the preparation of carbamoyl-*N*-aryl-imine-urea derivatives (9a–e and 10a–e) (adapted from ref. 19)

Under intermediate 15 or 16 (0.1 g or 0.3 mmol), was added at room temperature 10 mL of ethanol and 0.43 mmol (1 eq.) of selected aldehyde, followed by 1 drop of concentrated hydrochloric acid. A solution remained under stirring for 1–4 hours when TLC (dichloromethane : 5–10% methanol) indicated the completion of reaction. The volume of the reaction was reduced under reduced pressure (2/3 of volume), and after addition of ice was observed precipitation of the product which was vacuum filtered and washed with cold water.

##### 7.1. Preparation of compound 4-chloro-phenylcarbamoyl-*N*-5-nitrofuranimine-urea (compound 9a; LASSBio-1491)

Yield: 87%, yellow solid, mp 217–220 °C; IR (KBr) (cm^−1^): 3246 (*ν*NH); 1659 (*ν*C=O); 1504 (*ν*C=O); 1596 and 1351 (*δ* Ar-NO_2_); 1015 (*δ* Ar-Cl); ^1^H-NMR (200 MHz, DMSO-d_6_) *δ* (ppm): 11.24 (s, 1H, Ar-CONH); 10.39 (s, 1H, NHCONH); 9.12 (s, 1H, NHCONH); 7.93–7.89 (m, 3H, N=CH, H2, H6); 7.80 (d, 1H, H3, *J* = 4 Hz); 7.58 (d, 2H, H3, H5, *J* = 8 Hz); 7.31 (d, 1H, H4, *J* = 4 Hz); ^13^C-NMR (50 MHz. DMSO-d_6_) *δ* (ppm): 165.3 (Ar-CONH); 154.5 (C1′); 152.8 (C2′); 151.4 (NHCONH); 136.6 (C=NH); 131.3 (C4); 129.3 (C2, C6); 129.2 (C1); 128.6 (C3, C5); 115.1 (C3′); 112.6 (C4′); 99% purity in HPLC (R.T. = 4.98 min, CH_3_CN : H_2_O (1 : 1), *λ* = 370 nm); HRMS calculated for C_13_H_10_ClN_5_O_5_: [M + H]^+^ = 352.04432, found: *m*/*z* = 352.04417.

##### 7.2. Preparation of compound 4-chloro-phenylcarbamoyl-*N*-2-hydroxyphenylimine-urea (compound 9b; LASSBio-1704)

Yield: 53%, white solid, mp >250 °C; IR (KBr) (cm^−1^): 3309 (*ν*NH); 1701 and 1545 (*ν*C=O); 1664 and 1484 (*δ N*–H); 1011 (*δ* Ar-Cl); ^1^H-NMR (200 MHz, DMSO-d_6_) *δ* (ppm): 10.65 (s, 1H, Ar-CONH); 10.30 (s, 1H, NHCONH); 10.02 (s, 1H, OH); 8.98 (s, 1H, NHCONH); 8.22 (s, 1H, N=CH); 7.89 (t, 3H, H2, H6, *J* = 8 Hz, H2′); 7.57 (d, 2H, H3, H5, *J* = 8 Hz); 7.17 (t, 1H, H4′, *J* = 6 Hz); 6.88–6.81 (m, 2H, H3′, H5′); ^13^C-NMR (50 MHz. DMSO-d_6_) *δ* (ppm): 165.2 (Ar-CONH), 156.0 (C6′); 155.1 (NHCONH); 139.3 (C=NH); 136.5 (C4); 131.5 (C1); 130.4 (C4′); 129.3 (C2, C6); 128.5 (C3, C5); 127.1 (C2′); 120.2 (C1′); 119.1 (C3′), 116.0 (C5′); 99% purity in HPLC (R.T. = 4.86 min, CH_3_CN : H_2_O (4 : 6), *λ* = 320 nm). HRMS calculated for C1_5_H_13_ClN_4_O_3_: [M + H]^+^ = 333.07489, found: *m*/*z* = 333.07432.

##### 7.3. Preparation of compound 4-chloro-phenylcarbamoyl-*N*-3-hydroxyphenylimine-urea (compound 9c; LASSBio-1709)

Yield: 91%, white solid, mp 225–227 °C; IR (KBr) (cm^−1^): 3342 (*ν*NH); 1692 (*ν*C=O); 1655 (*ν*C=O); 1015 (*ν*Ar-Cl); ^1^H-NMR (200 MHz, DMSO-d_6_) *δ* (ppm): 10.62 (s, 1H, Ar-CONH); 10.28 (s, 1H, NHCONH); 9.47 (s, 1H, OH); 8.97 (s, 1H, NHCONH); 7.90 (d, 2H, H2, H6, *J* = 4 Hz); 7.80 (s, 1H, N=CH); 7.57 (d, 2H, H3, H5, *J* = 4 Hz); 7.17 (m, 3H, H2′, H3′, H6′); 6.77 (d, 1H, H4′, *J* = 4 Hz); 98% purity in HPLC (R.T. = 4.24 min, CH_3_CN : H_2_O (1 : 1), *λ* = 279 nm). HRMS calculated for C1_5_H_13_ClN_4_O_3_: [M + H]^+^ = 333.07489, found: *m*/*z* = 333.07407.

##### 7.4. Preparation of compound 4-chloro-phenylcarbamoyl-*N*-4-hydroxyphenylimine-urea (compound 9d; LASSBio-1706)

Yield: 86%, white solid, mp 223–225 °C; IR (KBr) (cm^−1^): 3369 (*ν*NH); 1701 (*ν*C=O); 1651 (*ν*C=O); 1014 (*ν*Ar-Cl); ^1^H-NMR (200 MHz, DMSO-d_6_) *δ* (ppm): 10.49 (s, 1H, Ar-CONH); 10.28 (s, 1H, NHCONH); 9.77 (s, 1H, OH); 8.92 (s, 1H, NHCONH); 7.90 (d, 2H, H2, H6, *J* = 8 Hz); 7.79 (s, 1H, N=CH); 7.59 (m, 4H, H3, H5, H2′, H6′); 6.77 (d, 2H, H3′, H5′, *J* = 8 Hz); ^13^C-NMR (50 MHz. DMSO-d_6_) *δ* (ppm): 165.2 (Ar-CONH); 158.7 (C4′); 155.3 (NHCONH); 141.2 (C=NH); 136.5 (C4); 131.5 (C1); 129.3 (C2, C6); 128.5 (C3, C5, C2′, C6′); 125.6 (C1′); 115.4 (C3′, C5′); 99% purity in HPLC (R.T. = 2.95 min, CH_3_CN : H_2_O (7 : 1), *λ* = 287 nm); HRMS calculated for C1_5_H_13_ClN_4_O_3_: [M + H]^+^ = 333.07489, found: *m*/*z* = 333.07455.

##### 7.5. Preparation of compound 4-chloro-phenylcarbamoyl-*N*-4-trifluoromethylphenylimine-urea (compound 9e; LASSBio-1737)

Yield: 85%, white solid, mp 201–203 °C; IR (KBr) (cm^−1^): 3338 (*ν*NH); 1701 (*ν*C=O); 1663 (*ν*C=O); 1015 (*ν*Ar-Cl); 1131 and 1100 (C–F); ^1^H-NMR (200 MHz, DMSO-d_6_) *δ* (ppm): 10.95 (s, 1H, Ar-CONH); 10.34 (s, 1H, NHCONH); 9.24 (s, 1H, NHCONH); 8.05 (d, 2H, H2, H6, *J* = 8 Hz); 7.96 (d, 2H, H2′, H6′, *J* = 8 Hz); 7.90 (s, 1H, N=CH); 7.75 (d, 2H, H3, H5, *J* = 8 Hz); 7.60 (d, 2H, H3′, H5′, *J* = 8 Hz); ^13^C-NMR (50 MHz. DMSO-d_6_) *δ* (ppm): 165.2 (Ar-CONH); 155.0 (NHCONH); 139.1 (N=CH); 138.5 (C4); 136.5 (C1′); 131.4 (C4′); 129.3 (C2, C6, C2′, C6′); 128.5 (C3, C5, C1); 127.3 (C3′, C5′); 125.3 (CF_3_); 99% purity in HPLC (R.T. = 5.95 min, CH_3_CN : H_2_O (6 : 1), *λ* = 320 nm). HRMS calculated for C_16_H_12_ClF_3_N_4_O_2_: [M + H]^+^ = 385.06736, found: *m*/*z* = 385.06695.

##### 7.6. Preparation of compound 2,4-dichloro-phenylcarbamoyl-*N*-5-nitrofuranimine-urea (compound 10a; LASSBio-1703)

Yield: 87%, yellow solid, mp 222–225 °C; IR (KBr) (cm^−1^): 3387 (*ν*NH); 1727 (*ν*C=O); 1667 (*ν*C=O); 1015 (*ν*C–Cl); 1244 and 1281 (N=O); ^1^H-NMR (200 MHz, DMSO-d_6_) *δ* (ppm): 11.22 (s, 1H, Ar-CONH); 10.22 (s, 1H, NHCONH); 9.18 (s, 1H, NHCONH); 7.86 (s, 1H, N=CH); 7.79 (d, 1H, H3′, *J* = 4 Hz); 7.71 (s, 1H, H5); 7.57 (m, 2H, H3, H6); 7.32 (d, 1H, H4′, *J* = 4 Hz); ^13^C-NMR (50 MHz. DMSO-d_6_) *δ* (ppm): 165.2 (Ar-CONH); 154.0 (NHCONH); 152.7 (C2′); 151.2 (C1′); 135.1 (C=NH); 133.3 (C4); 131.7 (C1); 130.7 (C2); 129.3 (C5); 129.1 (C6); 127.2 (C3); 115.0 (C3′); 112.5 (C4′); 99% purity in HPLC (R.T. = 3.41 min, CH_3_CN : H_2_O (7 : 1), *λ* = 370 nm); HRMS calculated for C_13_H_9_Cl_2_N_5_O_5_: [M + H]^+^ = 386.00535, found: *m*/*z* = 386.00500.

##### 7.7. Preparation of compound 2,4-dichloro-phenylcarbamoyl-*N*-2-hydroxyphenylimine-urea (compound 10b; LASSBio-1705)

Yield: 78%, white solid, mp 210–212 °C; IR (KBr) (cm^−1^): 3436–1852 (*ν*OH); 3232 (*ν*NH); 1708 (*ν*C=O); 1655 (*ν*C=O); 1046 (*ν*C–Cl); ^1^H-NMR (200 MHz, DMSO-d_6_) *δ* (ppm): 10.66 (s, 1H, Ar-CONH); 10.16 (s, 1H, NHCONH); 10.03 (s, 1H, OH); 9.11 (s, 1H, NHCONH); 8.23 (s, 1H, N=CH); 7.92 (d, 1H, H2′, *J* = 8 Hz); 7.73 (d, 1H, H5); 7.60 (m, 2H, H3, H6); 7.20 (t, 1H, H4′, *J* = 6 Hz); 6.86–6.79 (m, 2H, H3′, H5′); ^13^C-NMR (50 MHz. DMSO-d_6_) *δ* (ppm): 165.3 (Ar-CO); 155.9 (C6′); 154.8 (NHCONH); 139.1 (N=CH); 135.1 (C4); 133.5 (C1); 131.7 (C2); 130.8 (C4′); 130.5 (C3); 129.4 (C5); 127.3 (C6); 127.1 (C2′); 120.3 (C1′); 119.1 (C3′); 116.0 (C5′); 99% purity in HPLC (R.T. = 3.52 min, CH_3_CN : H_2_O (7 : 1), *λ* = 276 nm); HRMS calculated for C_15_H_12_Cl_2_N_4_O_3_: [M + H]^+^ = 367.03592, found: *m*/*z* = 367.03557.

##### 7.8. Preparation of compound 2,4-dichloro-phenylcarbamoyl-*N*-3-hydroxyphenylimine-urea (compound 10c; LASSBio-1708)

Yield: 96%, white solid, mp 223–225 °C; ^1^H-NMR (200 MHz, DMSO-d_6_) *δ* (ppm): 10.62 (s, 1H, Ar-CONH); 10.12 (s, 1H, NHCONH); 9.48 (s, 1H, OH); 9.05 (s, 1H, NHCONH); 7.80 (s, 1H, N=CH); 7.71 (s, 1H, H5); 7.70–7.50 (m, 2H, H3, H6); 7.19–16 (m, 3H, H2′, H3′, H6′); 6.76 (d, 1H, H4′, *J* = 8 Hz); 98% purity in HPLC (R.T. = 3.12 min, CH_3_CN : H_2_O (7 : 1), *λ* = 279 nm). HRMS calculated for C_15_H_12_Cl_2_N_4_O_3_: [M + H]^+^ = 367.03592, found: *m*/*z* = 367.03550.

##### 7.9. Preparation of compound 2,4-dichloro-phenylcarbamoyl-*N*-4-hydroxyphenylimine-urea (compound 10d; LASSBio-1707)

Yield: 90%, white solid, mp 225–227 °C; IR (KBr) (cm^−1^): 3362 (*ν*NH); 1694 (*ν*C=O); 1651 (*ν*C=O); 1098 (*ν*C–Cl); ^1^H-NMR (200 MHz, DMSO-d_6_) *δ* (ppm): 10.53 (s, 1H, Ar-CONH); 10.16 (s, 1H, NHCONH); 9.83 (s, 1H, OH); 9.05 (s, 1H, NHCONH); 7.84 (s, 1H, N=CH); 7.76 (s, 1H, H5); 7.67 (d, 2H, H2′, H6′, *J* = 10 Hz); 7.58–7.53 (m, 2H, H5, H6); 6.82 (d, 2H, H3′, H5′, *J* = 10 Hz); ^13^C-NMR (50 MHz. DMSO-d_6_) *δ* (ppm): 165.3 (Ar-CONH); 158.7 (C4′); 155.0 (NHCONH); 141.2 (N=CH); 135.1 (C4); 133.6 (C1); 131.7 (C2); 130.8 (C3); 129.4 (C5); 128.5 (C6); 127.3 (C1′); 125.5 (C2′, C6′); 115.4 (C3′, C5′); 99% purity in HPLC (R.T. = 3.05 min, CH_3_CN : H_2_O (7 : 1), *λ* = 287 nm); HRMS calculated for C_15_H_12_Cl_2_N_4_O_3_: [M + H]^+^ = 367.03592, found: *m*/*z* = 367.03563.

##### 7.10. Preparation of compound 2,4-dichloro-phenylcarbamoyl-*N*-4-trifluoromethylphenylimine-urea (compound 10e; LASSBio-1736)

Yield: 60%, white solid, mp 213–215 °C; IR (KBr) (cm^−1^): 3555 (*ν*NH); 1703 (*ν*C=O); 1655 (*ν*C=O); 1015 (*ν*C–Cl); 1164–1105 (C–F); ^1^H-NMR (200 MHz, DMSO-d_6_) *δ* (ppm): 10.95 (s, 1H, Ar-CONH); 10.17 (s, 1H, NHCONH); 9.33 (s, 1H, NHCONH); 8.09 (d, 2H, H2′, H6′, *J* = 10 Hz); 7.97 (s, 1H, C=NH); 7.77–7.74 (m, 3H, H3, H5, H6); 7.55 (m, 2H, H3′, H5′); ^13^C-NMR (50 MHz. DMSO-d_6_) *δ* (ppm): 165.3 (Ar-CONH); 154.7 (NHCONH); 139.1 (N=CH); 138.5 (C1′); 135.2 (C4); 133.5 (C1); 131.7 (C2′, C6′); 130.80 (C2); 129.4 (C3′, C5′); 129.3 (C5); 127.4 (C6, C3); 125.3 (C4′); 121.4 (CF_3_); 99% purity in HPLC (R.T. = 5.82 min, CH_3_CN : H_2_O (6 : 1), *λ* = 340 nm); HRMS calculated for C_16_H_11_Cl_2_F_3_N_4_O_2_: [M + H]^+^ = 419.02839, found: *m*/*z* = 419.02792.

#### 8 Plasma stability

For analysis of plasma stability, 5 μL (250 ng mL^−1^) of a concentrated solution of 10e (LASSBio-1736) or the positive control benzocaine (stock concentration = 10 000 ng mL^−1^ solubilized in DMSO) was added in 195 μL of rat plasma solution (1 : 1) diluted with PBS (pH 7.4). After vortexing the mixture was placed in a shaker at 37 °C under vigorous stirring for 0, 30, 60, 120 and 240 minutes. After each reaction time, 600 μL of cold methanol containing 100 ng mL^−1^ of ketoconazole (internal standard) were added to the wells to stop the reaction. The solution was mixed and centrifuged at 13 000 rpm for 15 min. The supernatant (400 μL) were placed in 2 mL vials, and analyzed by UPLC-MS/MS.

The analysis was performed using a Thermo Scientific Dionex UltiMate 3000 UHPLC liquid chromatography system (Thermo Fisher Scientific, Waltham, USA) coupled to a TSQ Quantiva Triple-Stage Quadrupole Mass Spectrometer, using electrospray ionization (ESI) and equipped with a degasser and TriPlus RSH autosampler (Thermo Fisher Scientific, Waltham, USA). A Syncronis column (Thermo, Waltham, USA; C_8_, 2.1 × 50 mm and 1.7 μM particle size) was employed and maintained at 40 °C. The mobile phase was prepared with Milli-Q water containing 0.1% formic acid and 5 mM ammonium formate (A) and with methanol containing 0.1% formic acid (B), and an 8 μL injection volume was used. Gradient elution at a flow rate of 0.3 mL min^−1^ was performed as follows: 1 min isocratic 90% A, decreased to 20% A at 10 min, and reduced to 0% A at 12.0 min through 14 min. The percentage of A was then increased to the initial condition of 90% at 14.1 min and was maintained until 16 min to equilibrate the column before the next injection. The total analysis time per sample was 16.0 min.

Detection in single reaction monitoring (SRM) mode was used for determination of the LASSBio-1736 (10e) *m*/*z* 419.1 → 205.0, benzocaine *m*/*z* 166.1 → 137.9 and ketoconazole (IS) 532.1 → 490.3, with a tolerance of mass deviation of 6 ppm, operating in positive ionization mode. The data were acquired using TraceFinder 3.2. software (Thermo Fisher Scientific, Waltham, USA), and analyzed using GraphPad Prism 5 program.

#### 9 Single crystal structure determination

The selected single crystal of compound 1491 was mounted and centered on a *κ*-goniostat and exposed to graphite-monochromated X-ray beam from Mo (Kα, *λ* = 0.71073 Å). An Enraf-Nonius Kappa-CCD diffractometer equipped with a CCD camera was employed to room temperature X-ray diffraction data collect. Data collection strategy was calculated by setting *φ* scans and *ω* scans with *κ* offsets. The crystallographic softwares were used as follows: Collect^23^ (X-ray diffraction experiment monitoring), HKL Denzo-Scalepack^26^ package of softwares (indexing, integration and scaling of raw data), SIR2004 (ref. 27) (structure solving), SHELXL-97 (ref. 28) (structure refinement), MERCURY^29^ and ORTEP-3 (ref. 30) (structure analysis and graphical representations). The structure was solved using the direct methods of phase retrieval. All non-hydrogen atoms of the asymmetric unit were directly located from the electronic density Fourier map. The early solved model was refined by full-matrix least squares method based on *F*.^26^ In the refinements, free anisotropic and isotropic thermal displacement parameters were adopted for non-hydrogen and hydrogen atoms, respectively. The isotropic thermal displacement parameters of hydrogens were 20% greater than the equivalent isotropic parameter of the bonded atom, except for water hydrogens which were refined freely. Concerning the positions of CH hydrogens, bond distances were stereochemically constrained according to riding model with lengths of 0.93 Å. All NH and water hydrogens had their coordinates refined freely. The main crystallographic experimental results are shown in Table S1.† CCDC 1824217 contains the supplementary crystallographic data for LASSBio-1491 (9a) crystal structure.

### Biology

#### 10 Parasite culture

Promastigotes of *L. amazonensis* (MHOM/BR/77/LTB0016) were obtained from Dr Eduardo Caio Torres dos Santos at Oswaldo Cruz Institute – Fiocruz. Promastigotes of *L. braziliensis* (MHOM/BR/01/BA788) were obtained from Dr Valéria de Matos Borges at Gonçalo Moniz Research Center – Fiocruz. The parasites were maintained *in vitro* in Schneider's medium, supplemented with 10% FBS and 2% human urine at 27 °C in BOD incubator.

#### 11 Culture of J774.A1 murine macrophages

These adherent-phenotype macrophage lines were cultured in Dulbecco's Modified Eagle's medium (DMEM, Sigma) supplemented with 10% FBS at 37 °C with 95% humidity and 5% CO_2_.

#### 12 Cytotoxicity against host cells

To evaluate the cytotoxic activity against the J774.A1 cell line, the host cells were plated in 96-well cell culture plate (flat bottom, clear, Corning) at 2 × 10^5^ cells per well in complete culture medium 10% FBS at 37 °C. After 1 h wells were washed with warm Hank's Balanced Salt solution (HBSS) to remove non-adherent cells, leaving approximately 10^5^ adherent macrophages. All cultures were done in DMEM complete supplemented with 10% FBS. The compounds and pentamidine were added at serial concentrations (0.1–100 μM). The cells were also cultured with medium free from compounds or vehicle (basal growth control) or in media with DMSO 0.1% (vehicle control). Positive control (dead cells) was obtained by cellular lysis with 1% of Triton 100× in DMEM complete (because it is a detergent in this concentration, Triton 100× destabilizes the cell membrane of macrophages, causing cell death). The cytotoxicity was evaluated by the MTT assay.^31^ This assay consists of indirectly measuring cell viability through the mitochondrial enzymatic activity of macrophages. Viable cells are capable of reducing MTT to formazan. For this, after the incubation of plate for 48 h, 100 μL of MTT solution (0.5 mg mL^−1^, in DMEM medium) was added per well, and the plate was incubated for 1 h. Then, the supernatant of each well was retired and was added 150 μL of DMSO. The absorbance was determined at 570 nm in a microplate reader. The cell viability of the cultures treated with the compounds was compared to the death pattern obtained in the control cultures. In the positive control, the cell death was confirmed when the optical density was equal to zero, confirming that the cells were not viable. Data obtained from experiments were expressed as the mean ± standard error of the mean (mean ± S.E.M.) and statistical differences between the treated and the vehicle groups of experiments were evaluated by ANOVA and Dunnett hoc tests.

#### 13 *In vitro* activity against amastigote forms of *Leishmania*

Initially, compounds 9a–e and 10a–e were evaluated at screening concentration of 30 μM against *L. amazonensis*, using DMSO 0.03% as vehicle control, since this was the DMSO concentration (vehicle) remaining in each well with the tested compounds. Compounds that exhibited activity ≥ 30% were selected and their leishmanicidal profile, in different concentrations (100, 10, 1, 10^−1^, 10^−2^ and 10^−3^ μM), was evaluated against *L. amazonensis* and *L. braziliensis*, in order to determine the IC_50_. Vehicle control used was DMSO 0.1%, since this was the concentration of DMSO (vehicle) existing in each well with the tested substances in the highest concentration used.

To assess the activity of the test derivatives against the amastigote stage of *L. amazonensis* and *L. braziliensis* the model of infection in coverglass was realized.^31^ The murine macrophages (J774.A1 cell line) were prepared in 24-well cell culture plate (flat bottom, clear, Corning) at 2 × 10^5^ adherent cells per well, infected with 2 × 10^6^ promastigotes in glass coverslips placed inside 1 mL medium culture. The cultures were cultured or not with the test derivatives or reference drugs, and kept for 24 h at 37 °C, 5% CO_2_. After 24 h of incubation, the coverslips were washed, stained with Giemsa–May–Grünwald, and intracellular amastigotes were counted in 100 macrophages, using an optical microscope, on the objective of 100×. Thus, during the counting of 100 macrophages, the amount of amastigotes present within these defence cells was also determined. Data obtained from *in vitro* experiments were expressed as the mean ± S.E.M. of duplicate cultures of representative assays. Statistical differences between the treated and the control groups were evaluated by ANOVA and Dunnett hoc tests. Differences with a *p* value < 0.05 or lower were considered significant.

#### 14 *In vivo* activity against *L. amazonensis*

This study (protocol number 2013.02) was approved by the Ethics Committee for Animal Experimentation of the Federal University of Alagoas (Brazil). All animals received humane care in compliance with the ‘Principles of laboratory animal care’ formulated by the National Society for Medical Research and the ‘Guide for the care and use of laboratory animals’ prepared by the National Academy of Sciences (Washington, DC). Then, 1 × 10^5^ stationary promastigotes (5 days of culture in Schneider's medium) of *L. amazonensis* were inoculated subcutaneously into the right ear dermis of 6 week-old female BALB/c mice weighing *ca.* 20 g. and later treated with 10e (i.p.), miltefosine (p.o.) or meglumine antimoniate (i.p.) at 30 μmol kg^−1^ × 28 days. The vehicle to solubilize 10e (LASSBio-1736) and standard drugs was Tween 20 (50 μL) and sterile water for injection, which was also administrated by i.p. route in two controls groups (non-infected control and infected control). The lesion size was measured using a paquimeter.^32^ The parasite loads of infected ears and draining lymph nodes were determined using a quantitative limiting-dilution assay.^33^ Toxicity was also evaluated due to alterations on spleen weight and by biochemistry dosages in plasma, performed according to the manufacturer's instructions (Doles, BRA). Data obtained from experiments were expressed as the mean ± S.E.M. and statistical differences between the treated and the vehicle groups of experiments were evaluated by ANOVA and Dunnett hoc tests.

## Experimental conclusions

A unique peptide mimetic framework was proposed for the first time based on molecular hybridization between amide and semicarbazone subunits. From this framework, a series of carbamoyl-*N*-aryl-imine-ureas were prepared and evaluated. Compound 10e (LASSBio-1736) was identified as a new antileishmanial drug candidate, displaying plasma stability, cytotoxicity against amastigote forms of *L. amazonensis* and *L. braziliensis*, and leishmanicidal activity in a cutaneous leishmaniasis murine model, without preliminary evidence of hepatic or renal toxicity.

These data also highlight perspectives for investigating the potential of 10e (LASSBio-1736) as a cysteine protease inhibitor, as well as encouraging the extension of these studies to determine the mechanism of induction of parasite death and perform LASSBio-1736 (10e) structure optimization.

## Conflicts of interest

There are no conflicts to declare.

## Supplementary Material

RA-010-D0RA00287A-s001

RA-010-D0RA00287A-s002
