# Supplementary material for: Carbamoyl-N-aryl-imine-urea: a new framework to obtain a putative leishmanicidal drug-candidate
Source: RSC Adv. 2020 Mar 26;10(21):12384–94. doi: 10.1039/d0ra00287a (PMC9050848; doi:10.1039/d0ra00287a)
Supplement: RA-010-D0RA00287A-s001 [file RA-010-D0RA00287A-s001.pdf]

Electronic Supplementary Material (ESI) for RSC Advances.
This journal is © The Royal Society of Chemistry 2020

Supporting Information

Carbamoyl-*N*-aryl-imine-urea: A new framework to obtain a putative leishmanicidal drug-candidate

Marina A. Alves[1,2]‡, Aline C. de Queiroz[3]‡, Anderson Brandão Leite[3], Felipe T. Martins[4], Antonio C. Doriguetto[4], Eliezer J. Barreiro[1,2], Magna S. Alexandre-Moreira[3], Lídia M. Lima[1,2]*

Contents of SI

1 – ^{1}H and ^{13}C NMR Spectra, IR Spectra and HRMS analyses of Carbamoyl-*N*-aryl-imine-urea (9a-e)

2 – ^{1}H and ^{13}C NMR Spectra, IR Spectra and HRMS analyses of Carbamoyl-*N*-aryl-imine-urea (10a-e)

3- Table S1. Crystal data and refinement parameters of compound 9a (LASSBio-1491).

4- Chemical stability pH 7.4

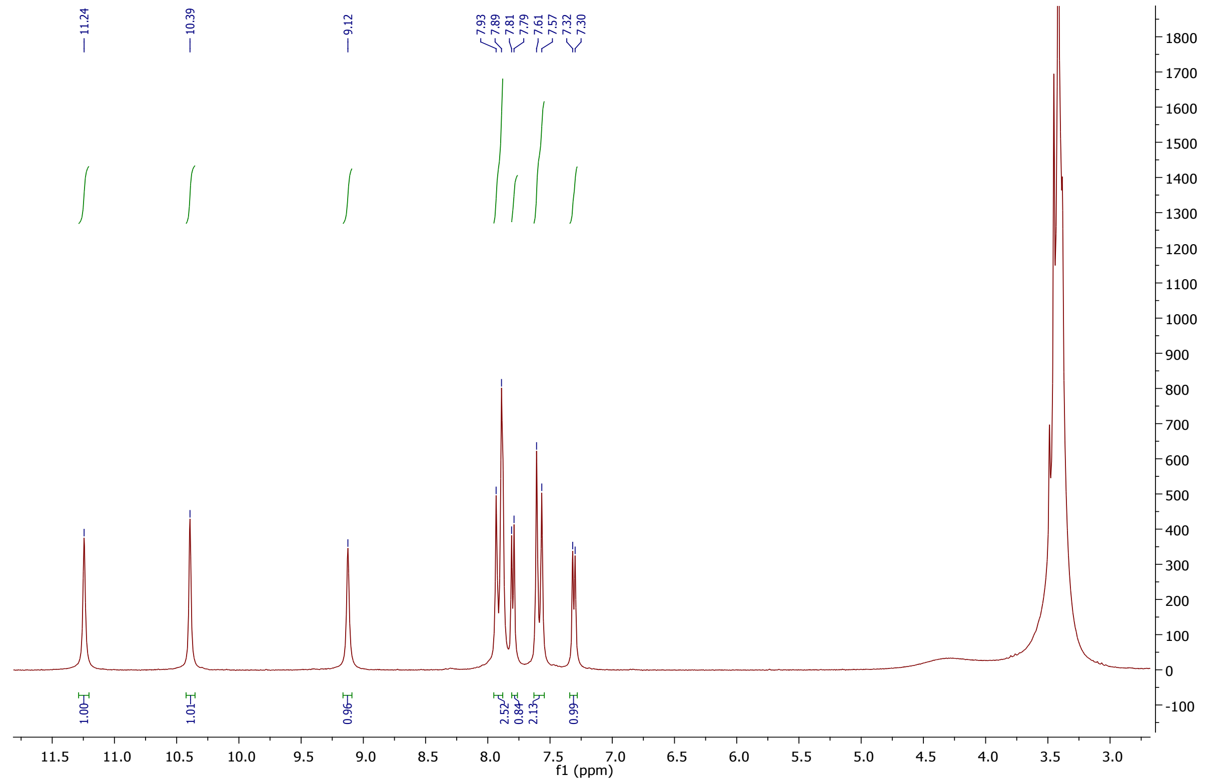

Figure S1. Compound 9a (LASSBio-1491) ^{1}H NMR

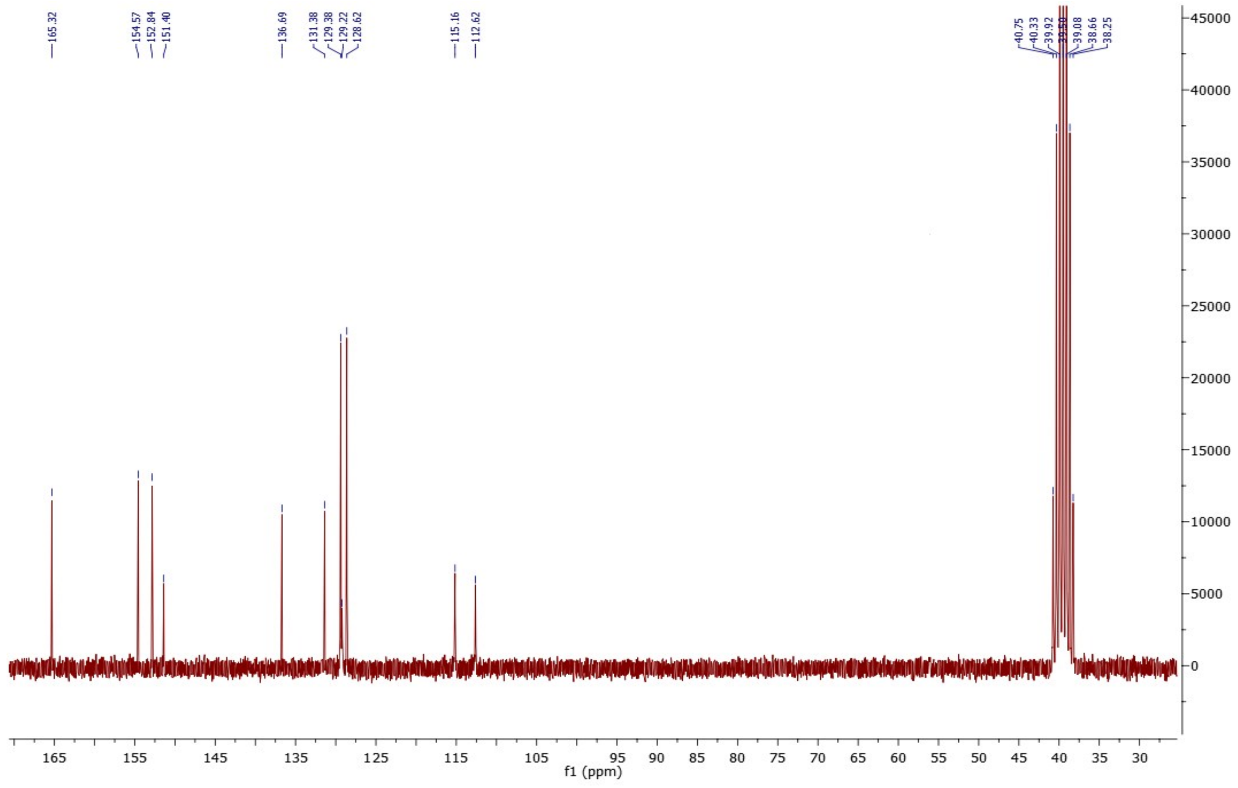

Figure S2. Compound 9a (LASSBio-1491) ^{13}C NMR

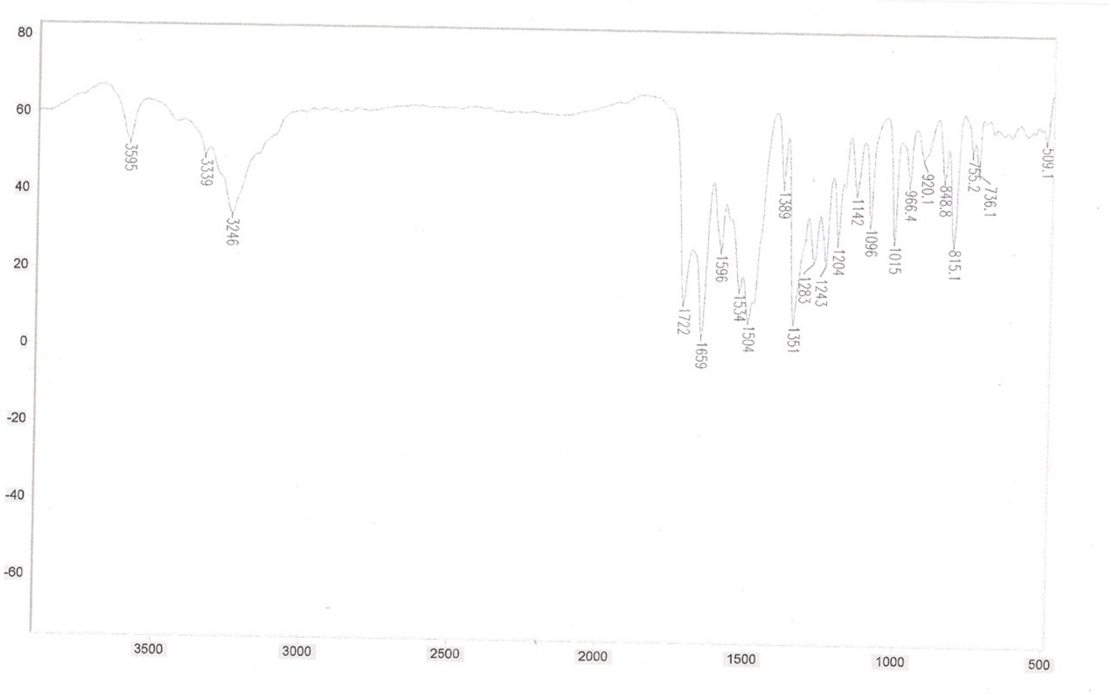

Figure S3. Compound 9a (LASSBio-1491) IR

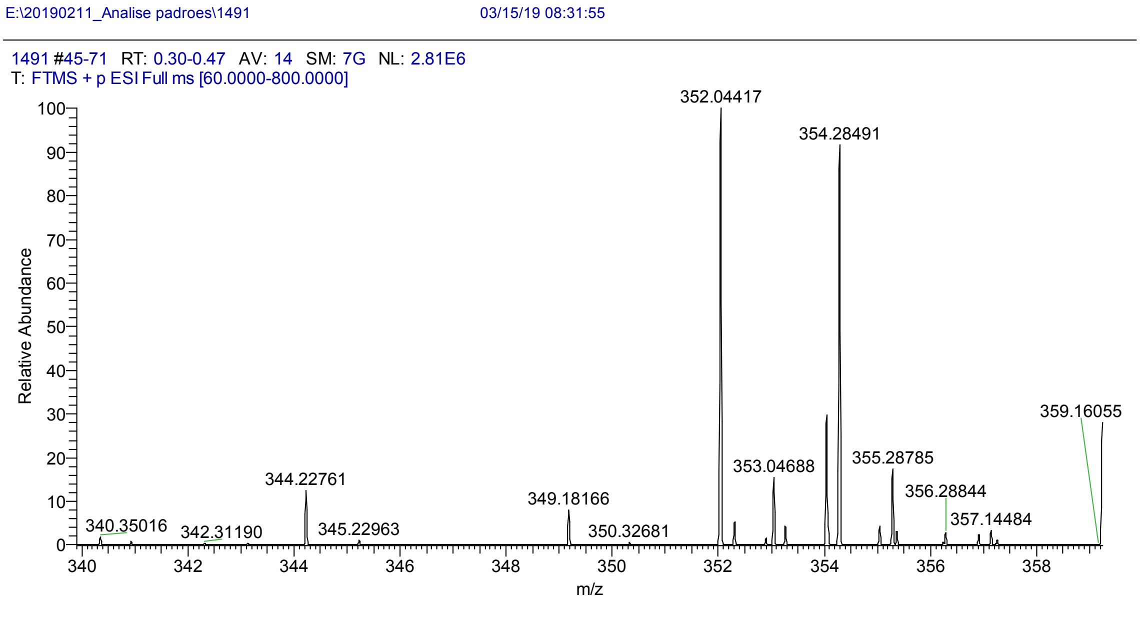

Figure S4. Compound 9a (LASSBio-1491) HRMS

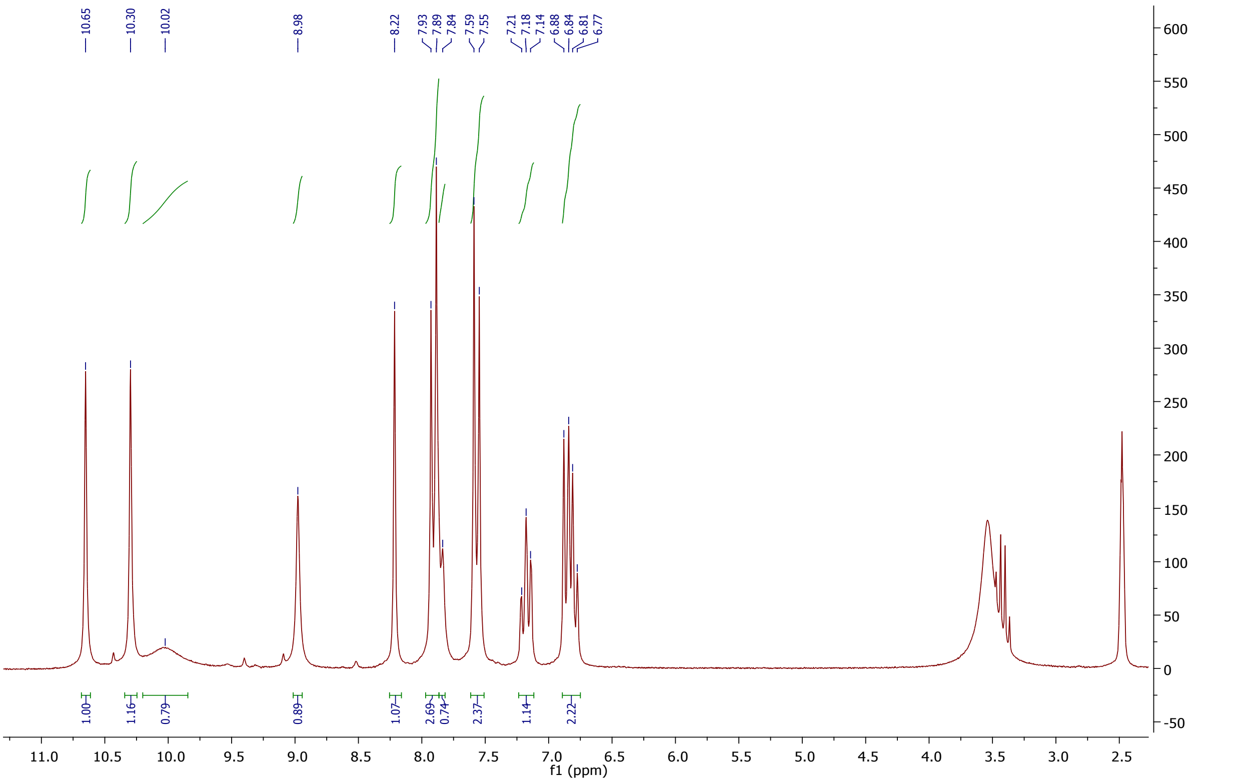

Figure S5. Compound 9b (LASSBio-1704) ^{1}H NMR

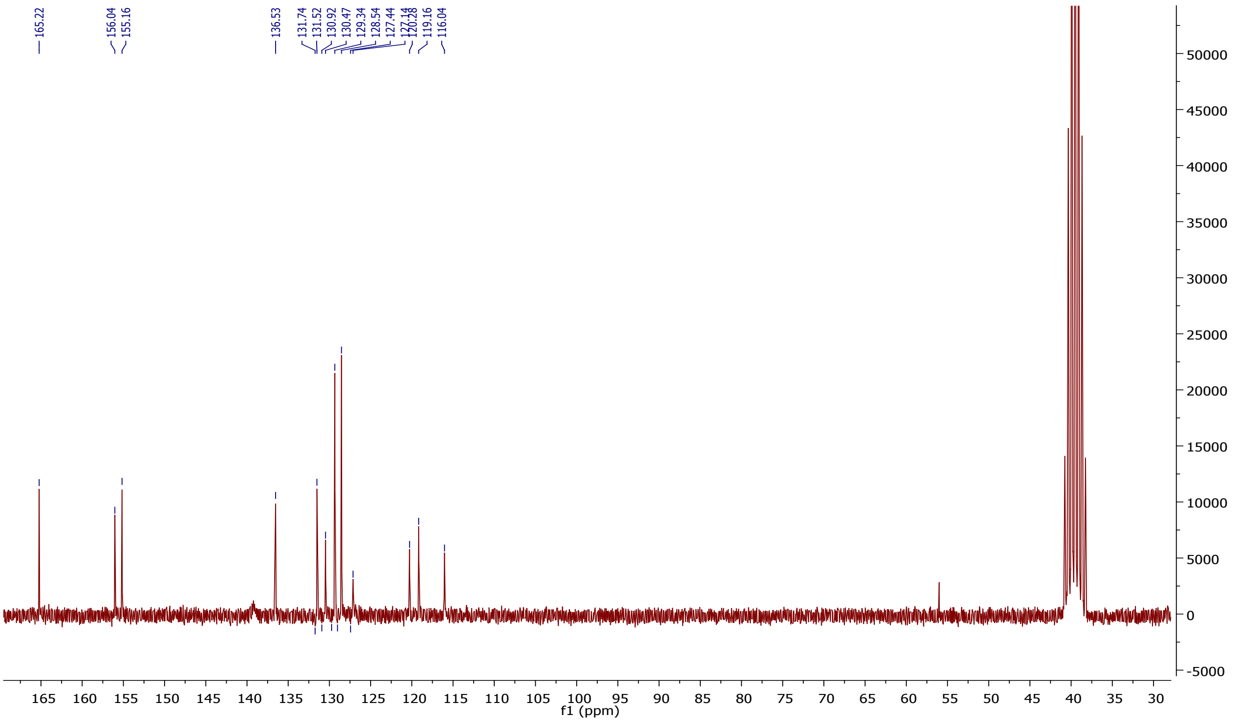

Figure S6. Compound 9b (LASSBio-1704) ^{13}C NMR

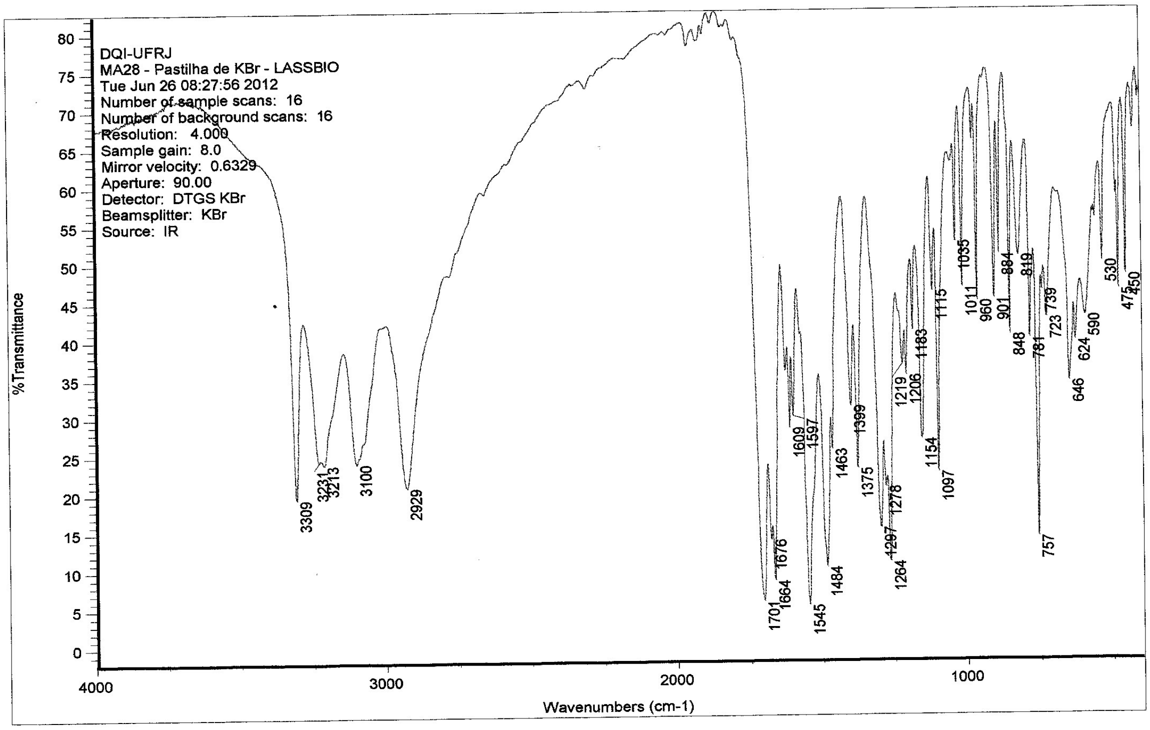

Figure S7. Compound 9b (LASSBio-1704) IR

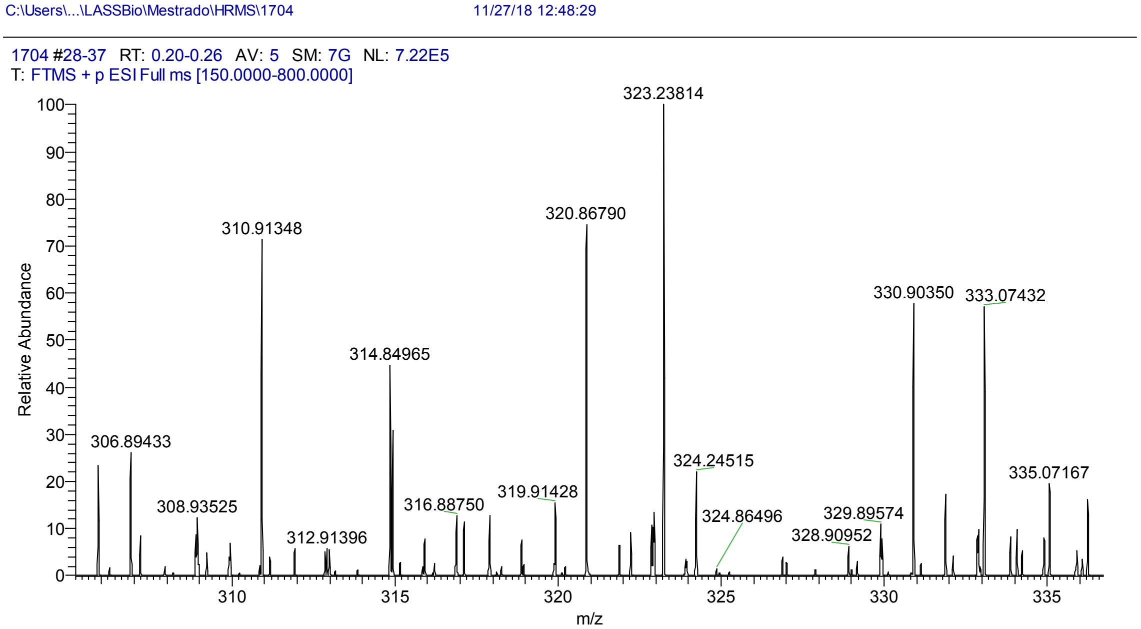

Figure S8. Compound 9b (LASSBio-1704) HRMS

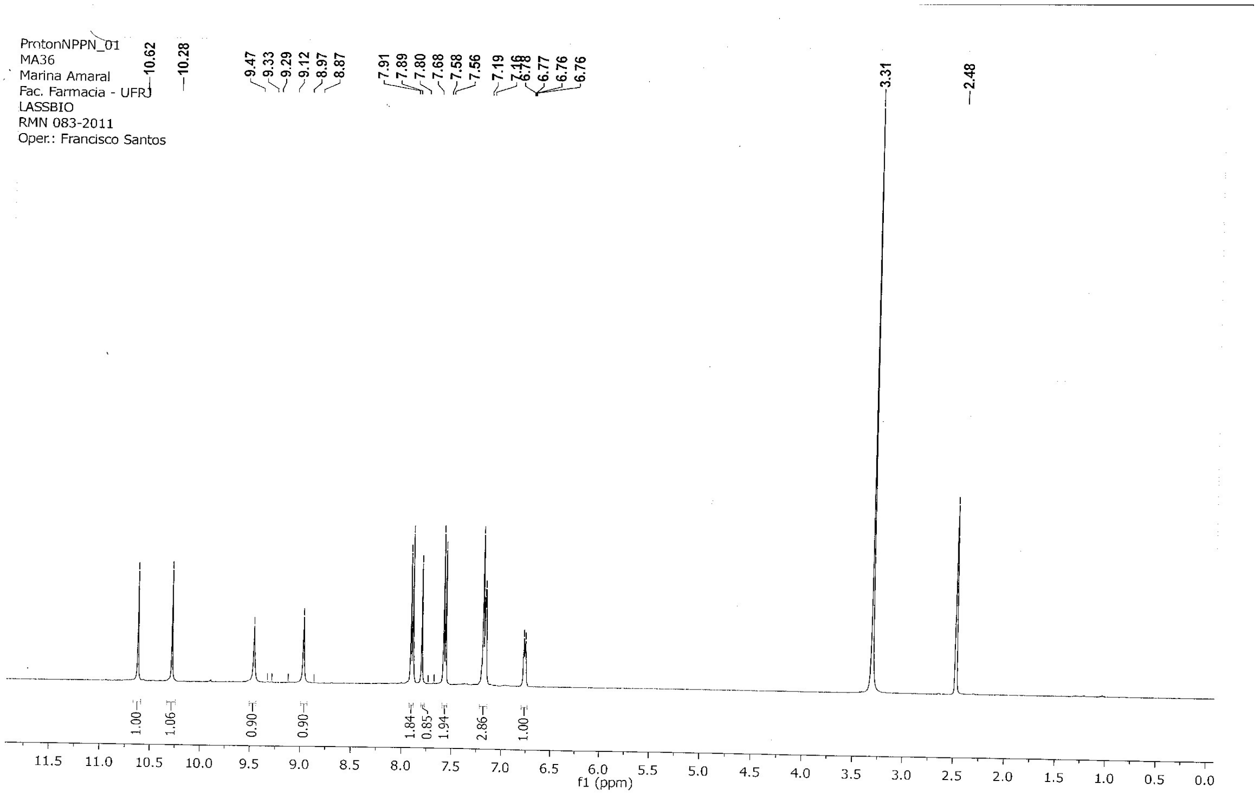

Figure S9. Compound 9c (LASSBio-1709) ^1H NMR

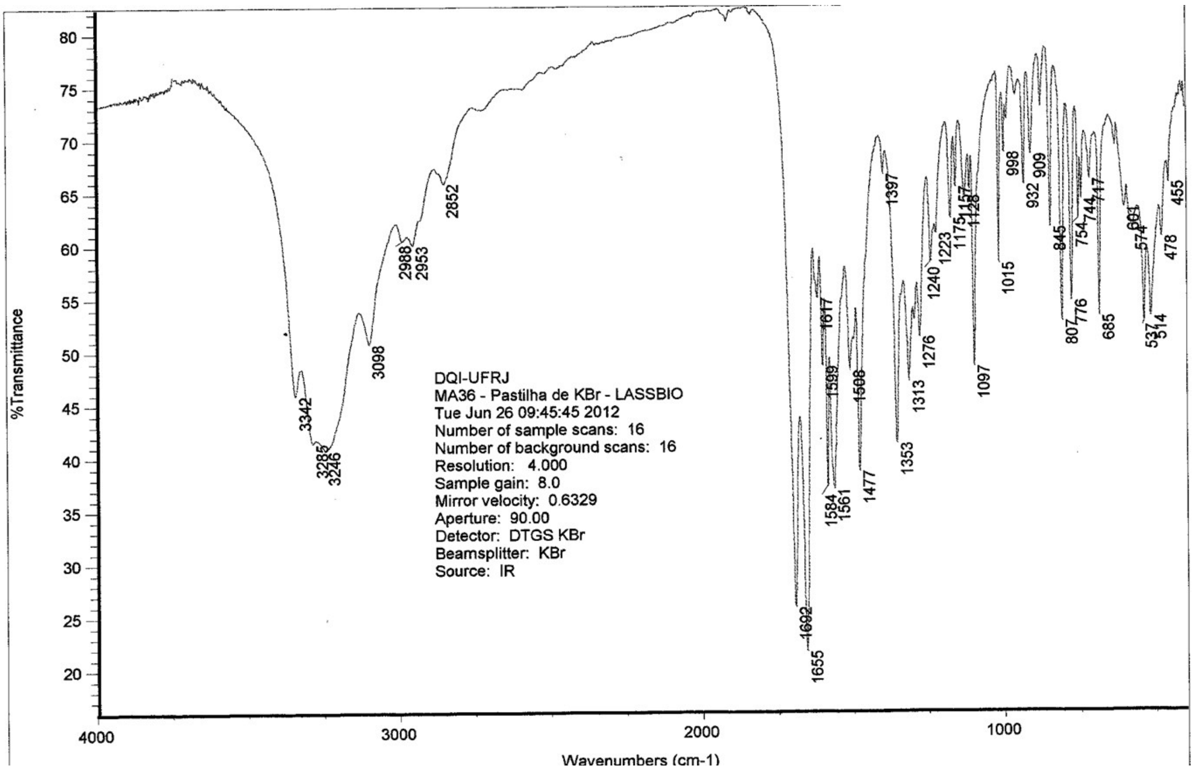

Figure S10. Compound 9c (LASSBio-1709) IR

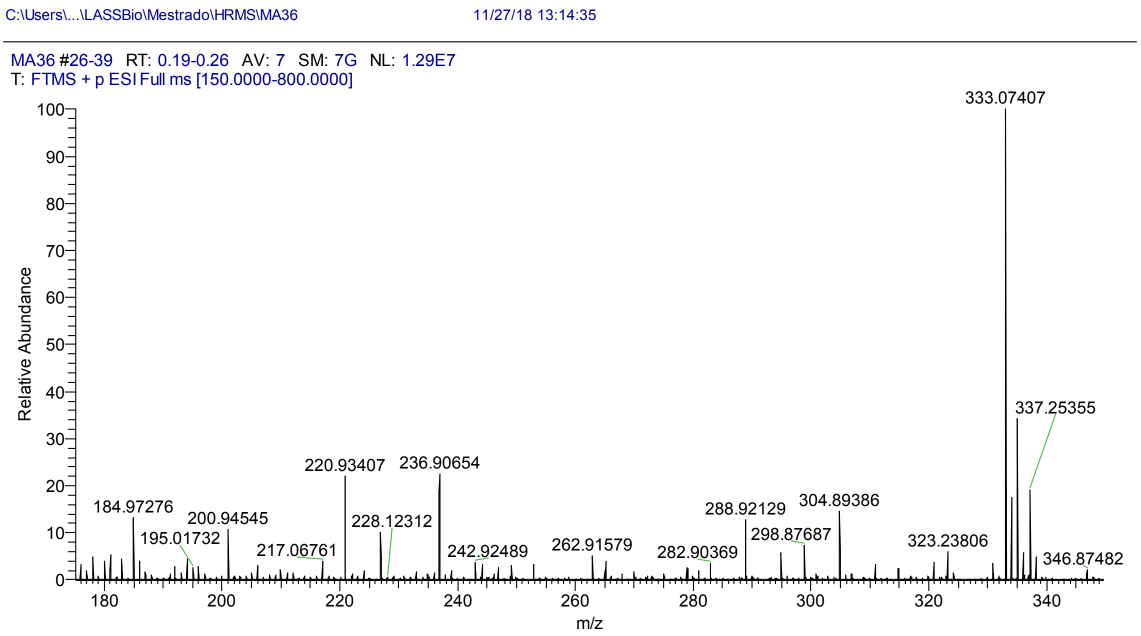

Figure S11. Compound 9c (LASSBio-1709) HRMS

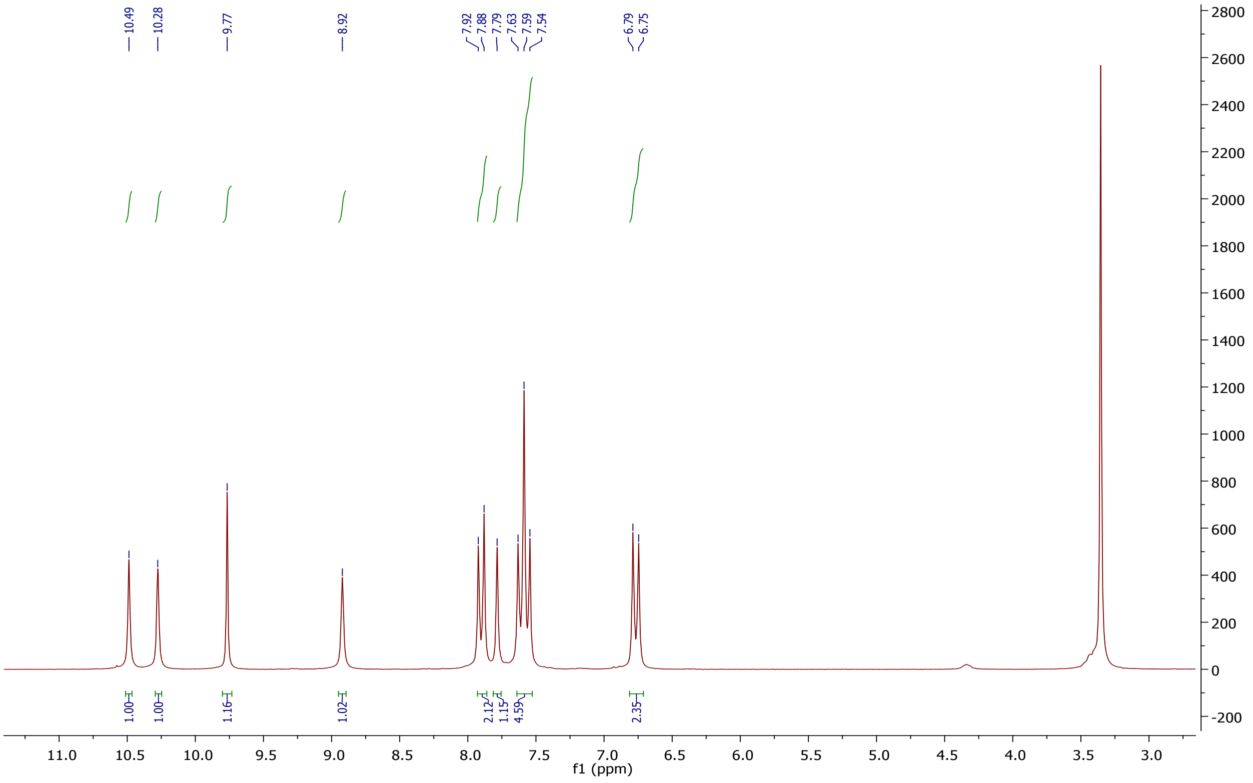

Figure S12. Compound 9d (LASSBio-1706) ^{1}H NMR

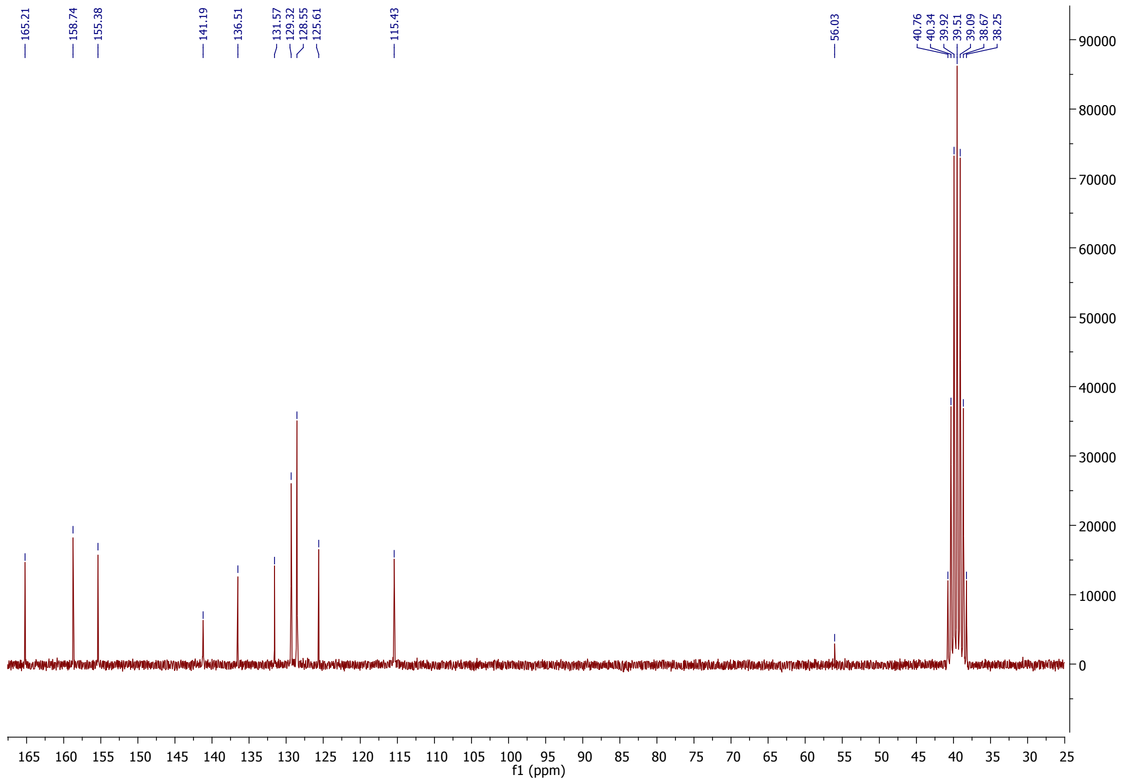

Figure S13. Compound 9d (LASSBio-1706) ^{13}C NMR

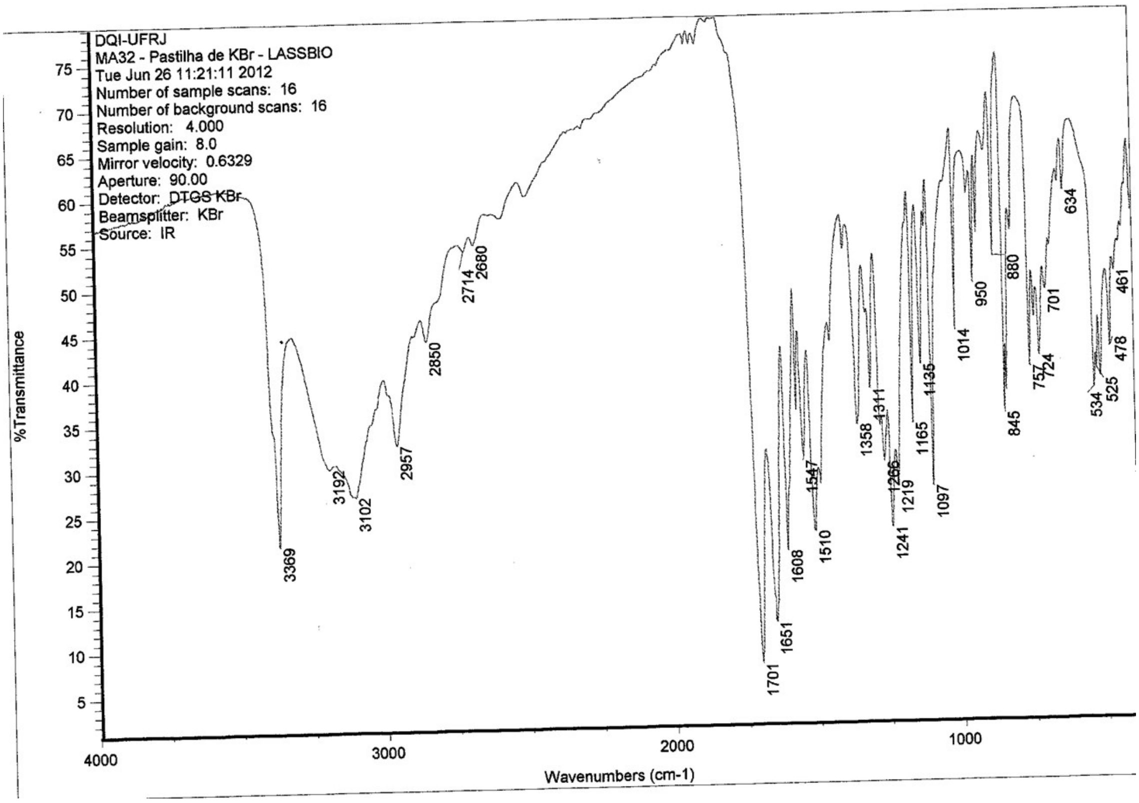

Figure S14. Compound 9d (LASSBio-1706) ^{13}C NMR

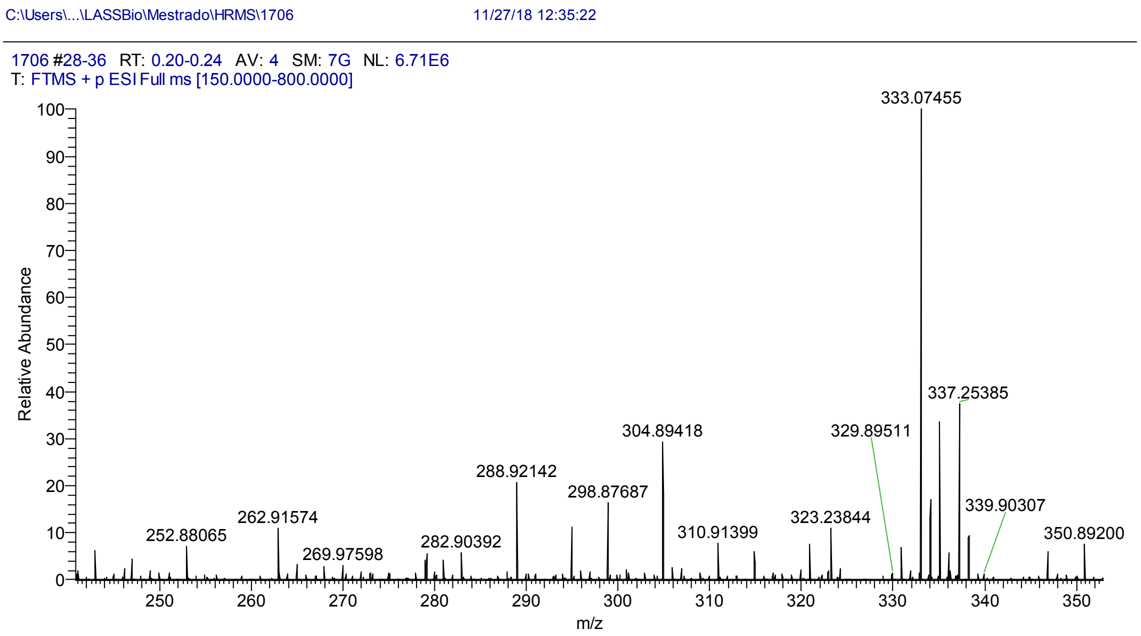

Figure S15. Compound 9d (LASSBio-1706) HRMS

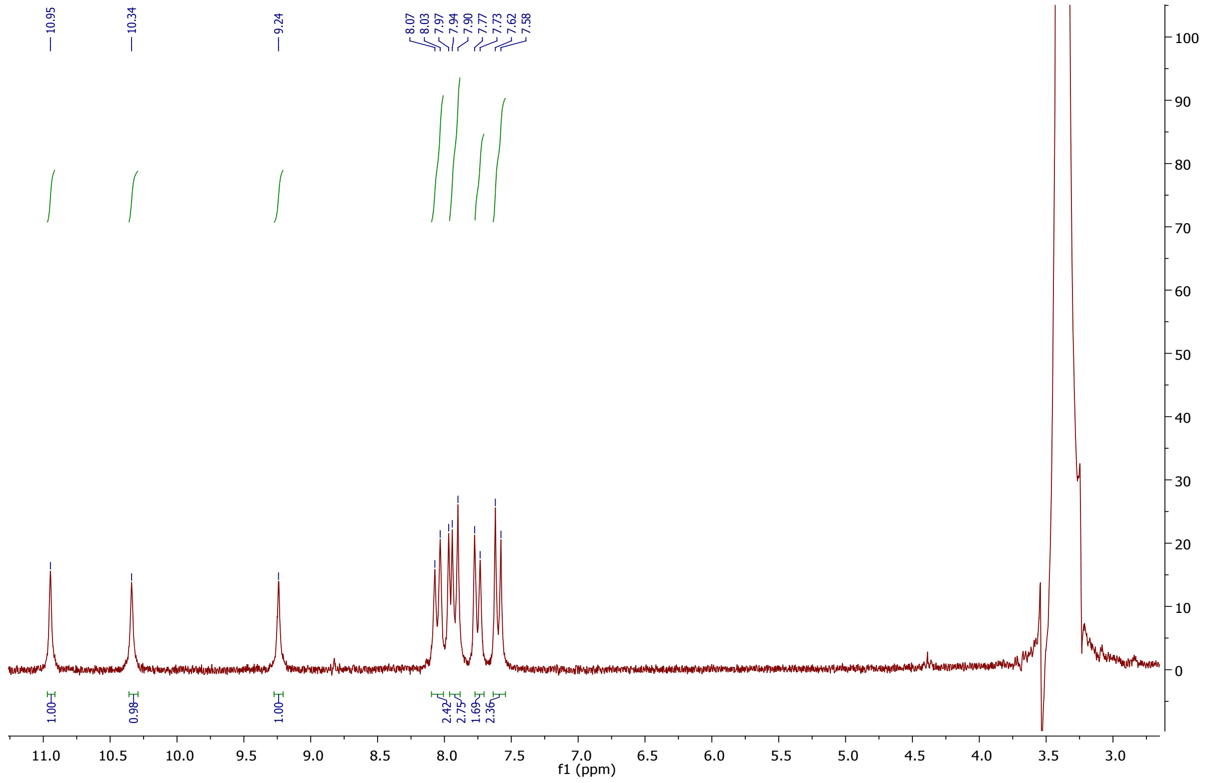

Figure S16. Compound 9e (LASSBio-1737) ^{1}H NMR

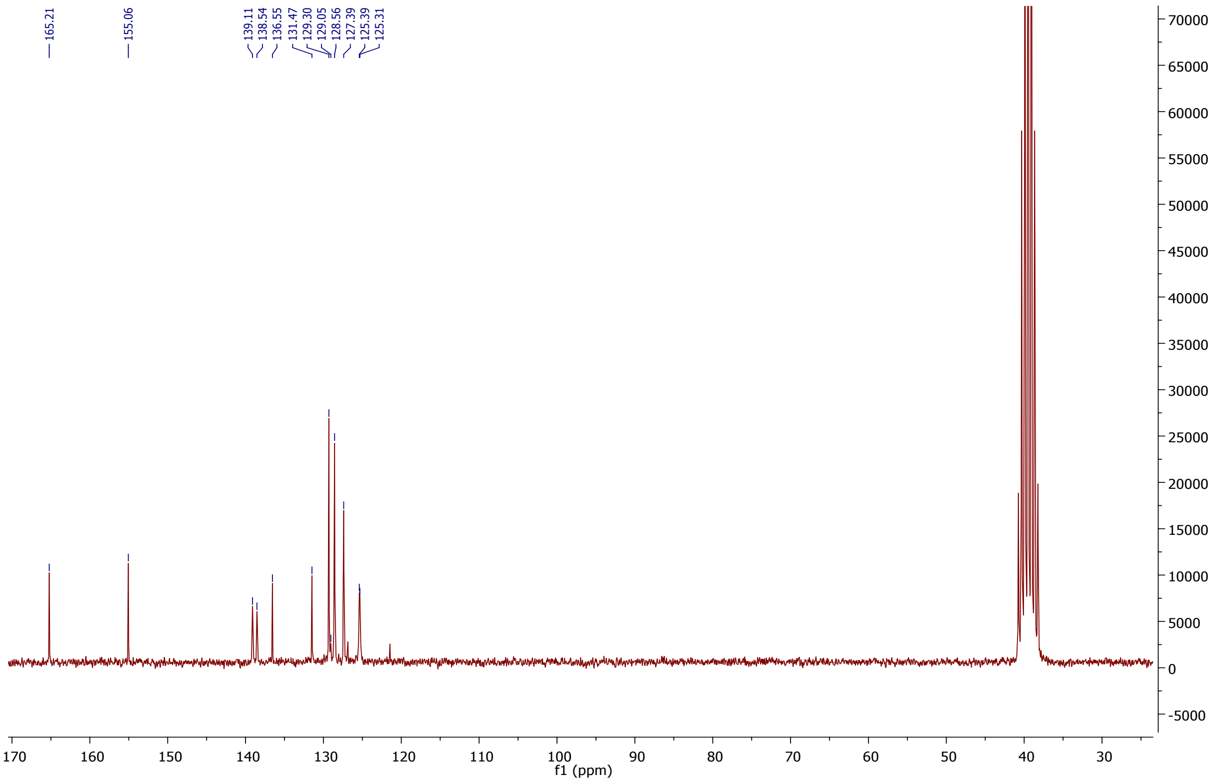

Figure S17. Compound 9e (LASSBio-1737) ^{13}C NMR

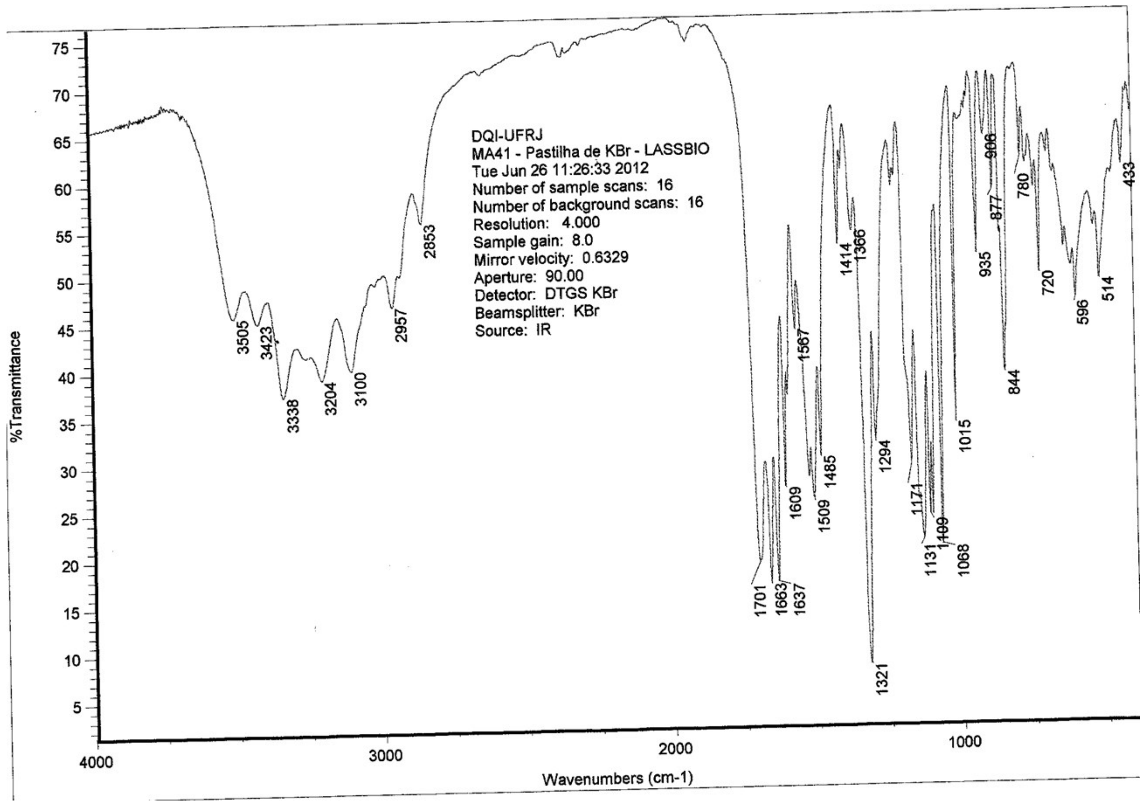

Figure S18. Compound 9e (LASSBio-1737) IR

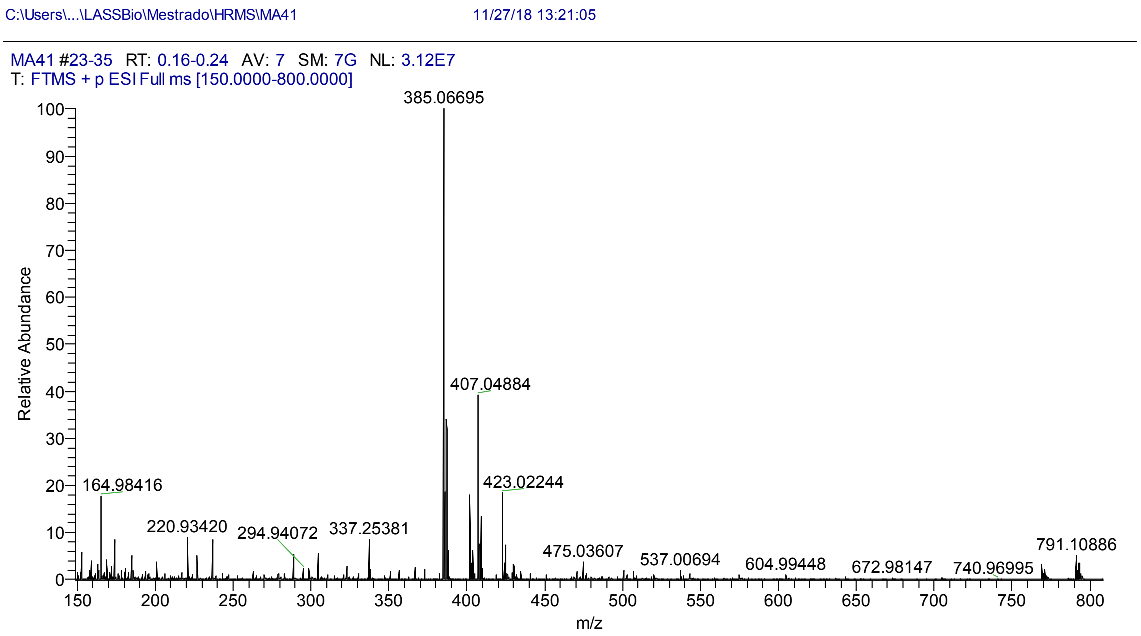

Figure S19. Compound 9e (LASSBio-1737) HRMS

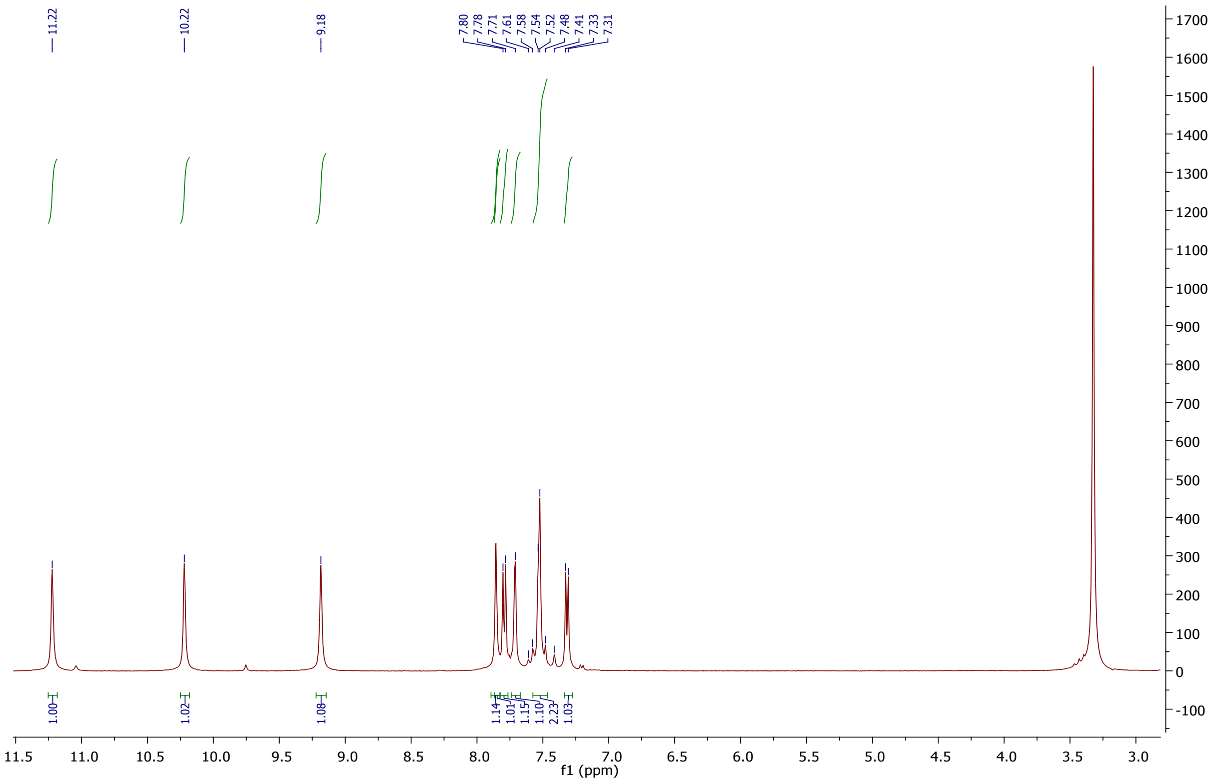

Figure S20. Compound 10a (LASSBio-1703) ^{1}H NMR

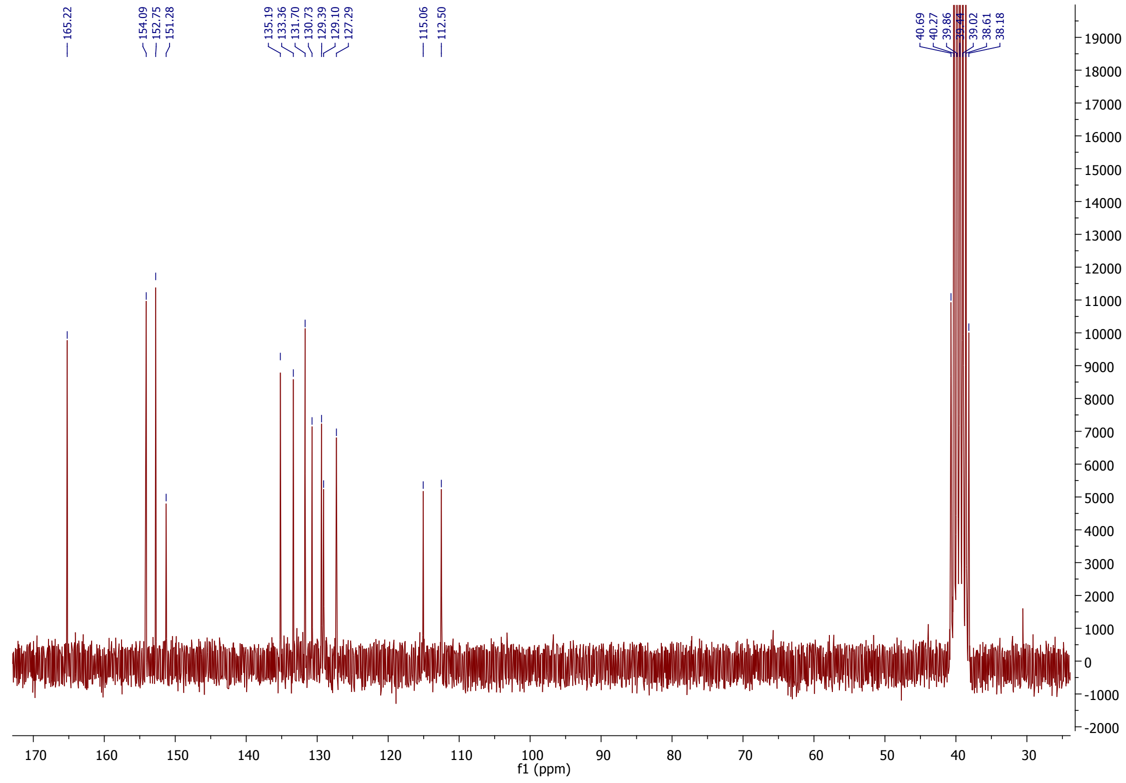

Figure S21. Compound 10a (LASSBio-1703) ^{13}C NMR

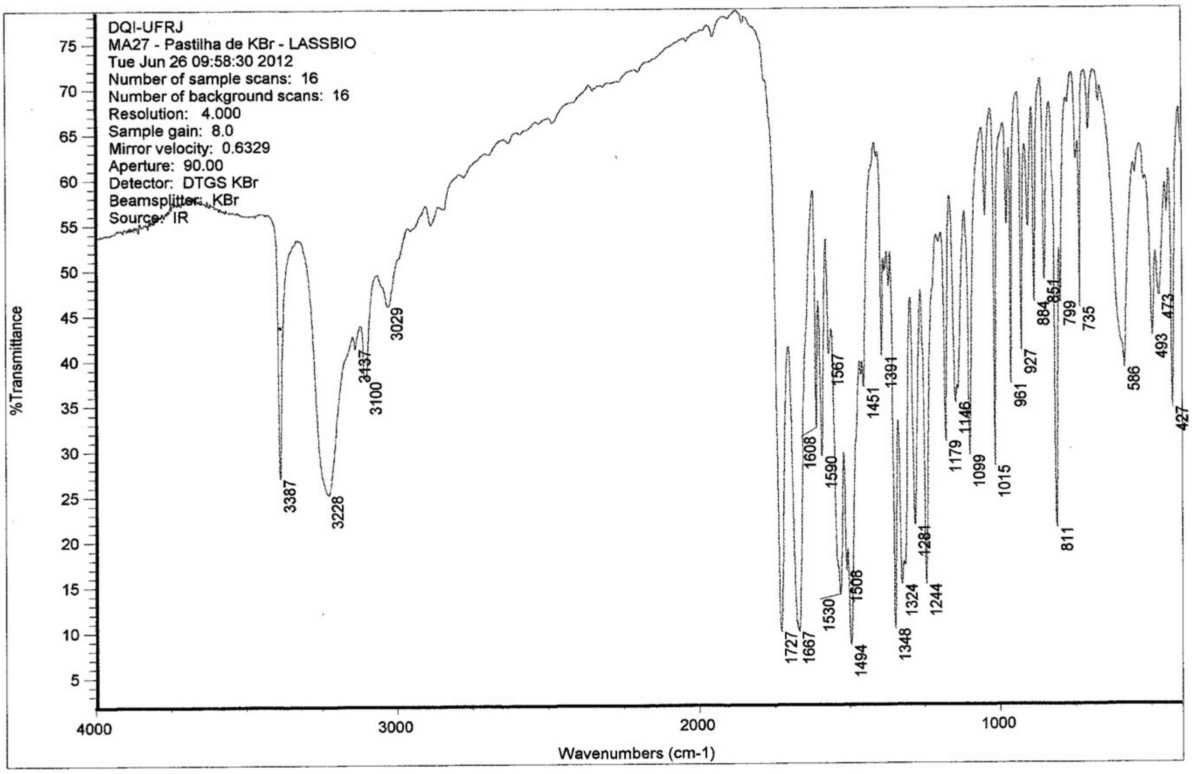

Figure S22. Compound 10a (LASSBio-1703) IR

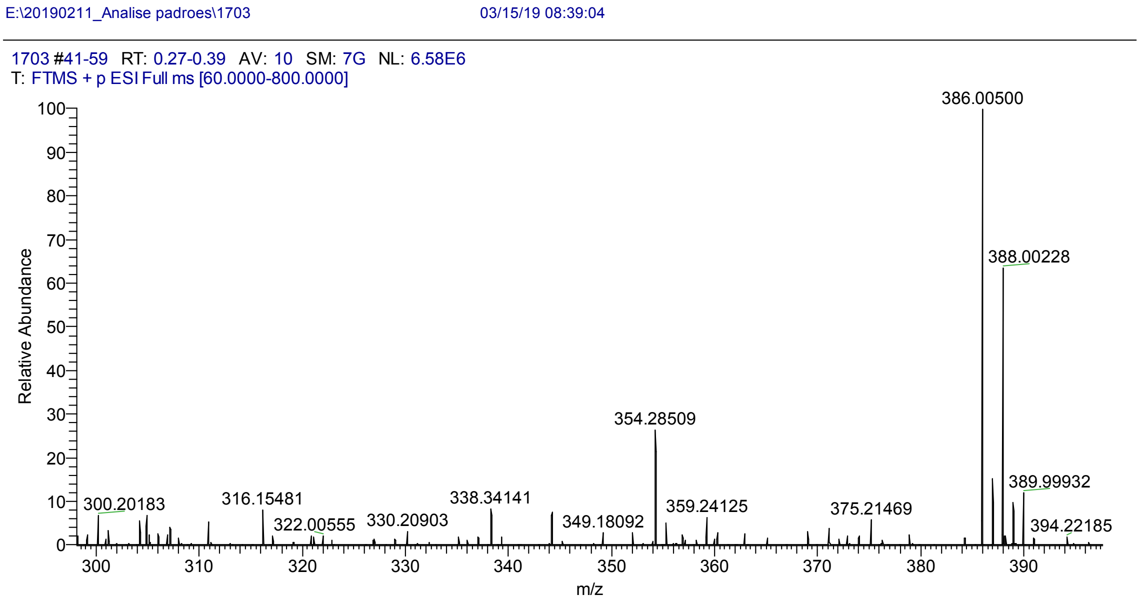

Figure S23. Compound 10a (LASSBio-1703) HRMS

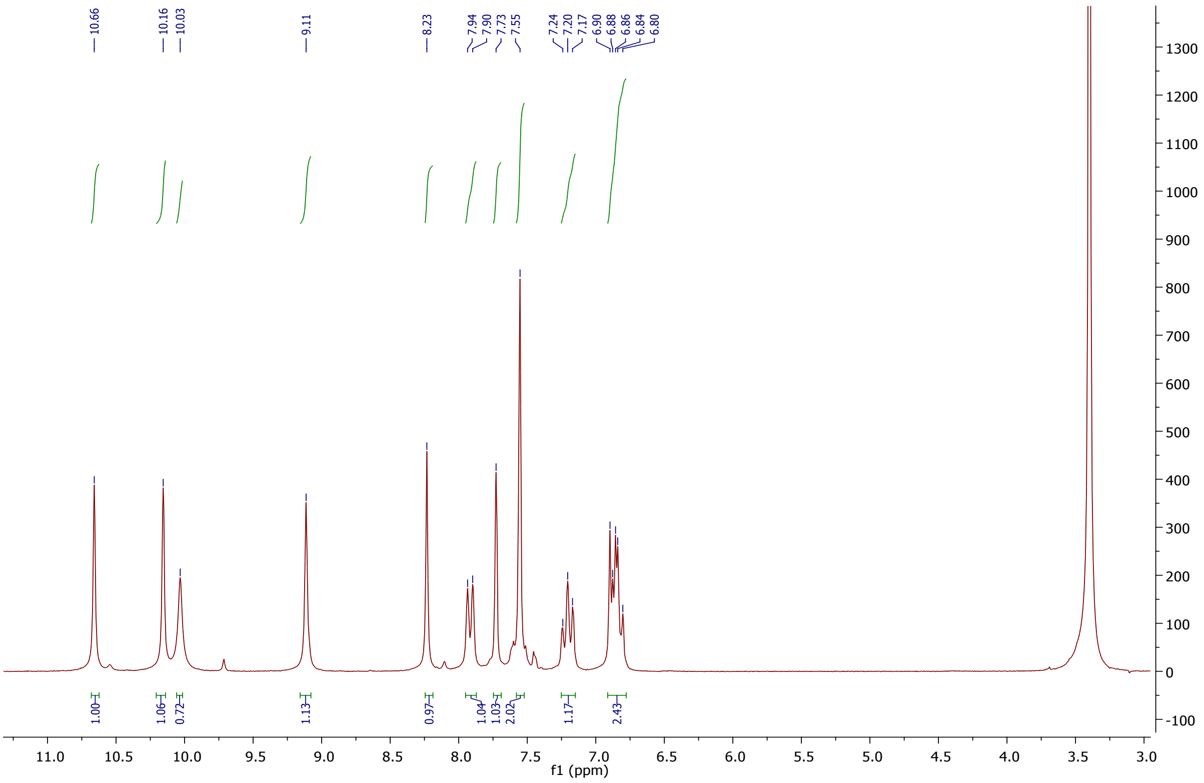

Figure S24. Compound 10b (LASSBio-1705) ^{1}H NMR

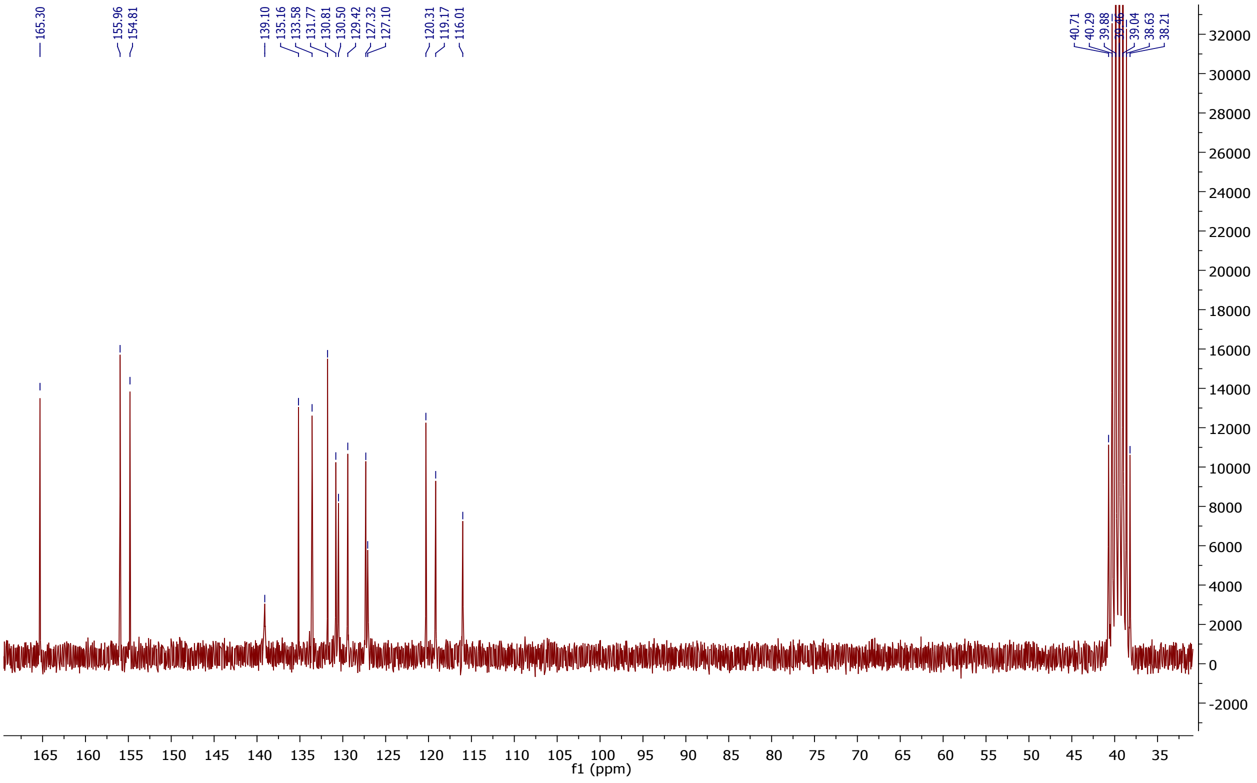

Figure S25. Compound 10b (LASSBio-1705) ^{13}C NMR

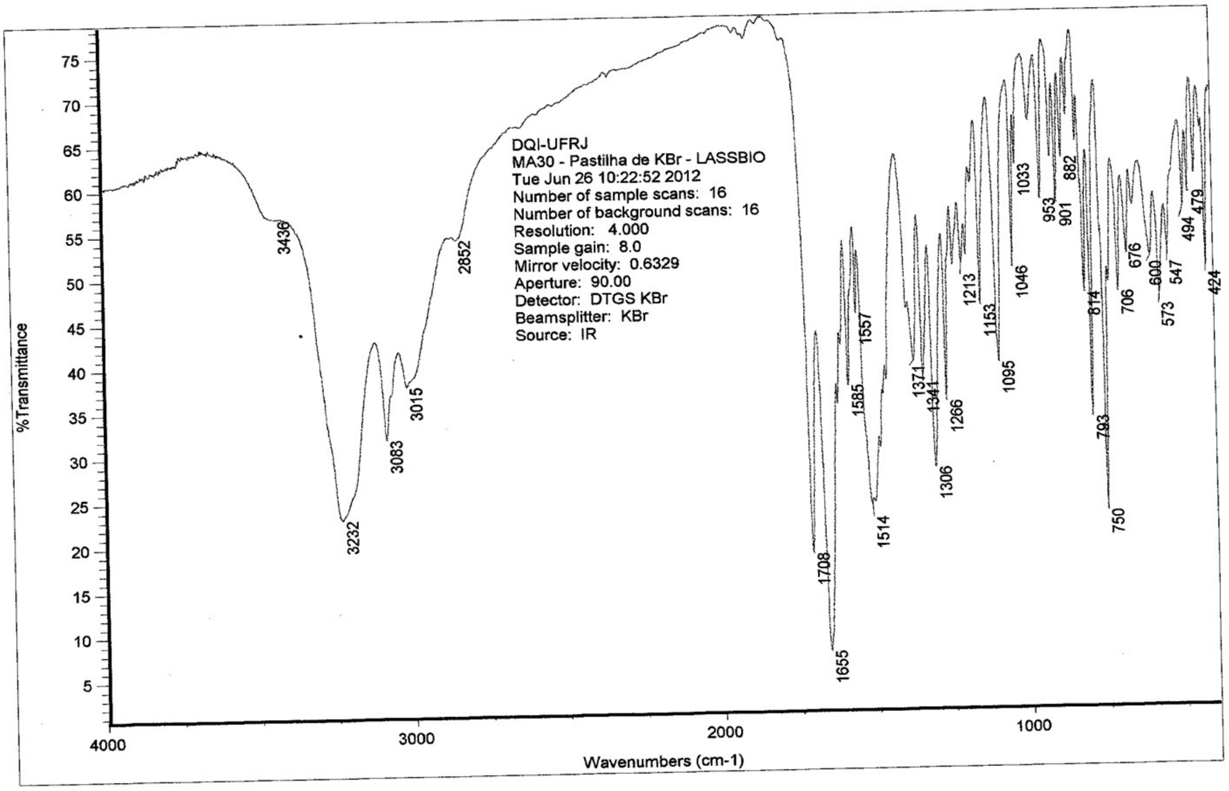

Figure S26. Compound 10b (LASSBio-1705) IR

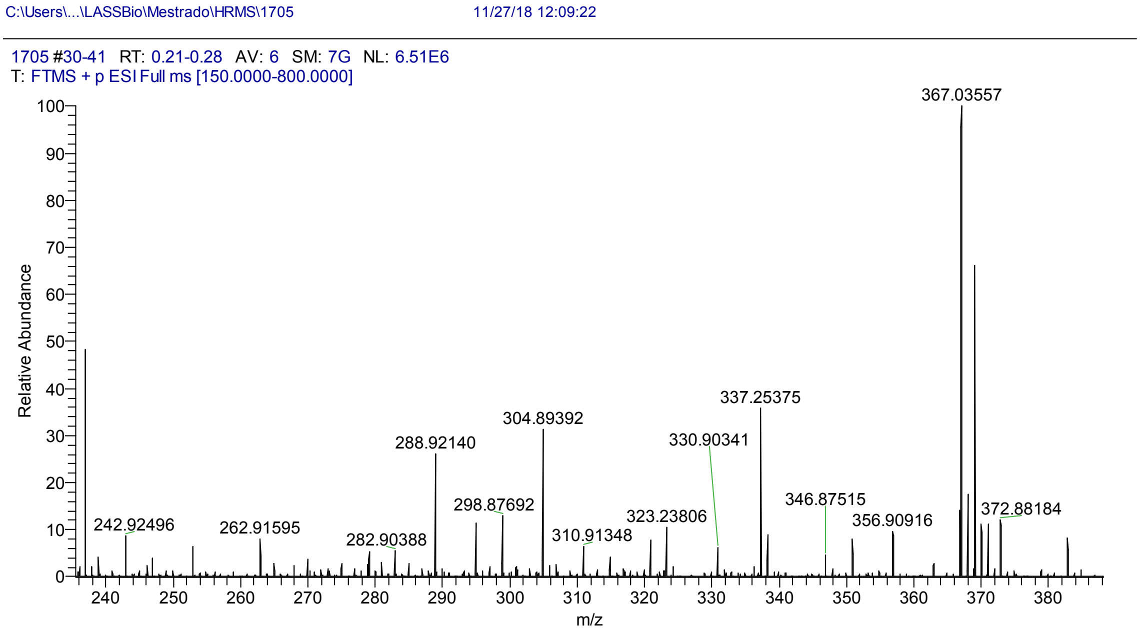

Figure S27. Compound 10b (LASSBio-1705) HRMS

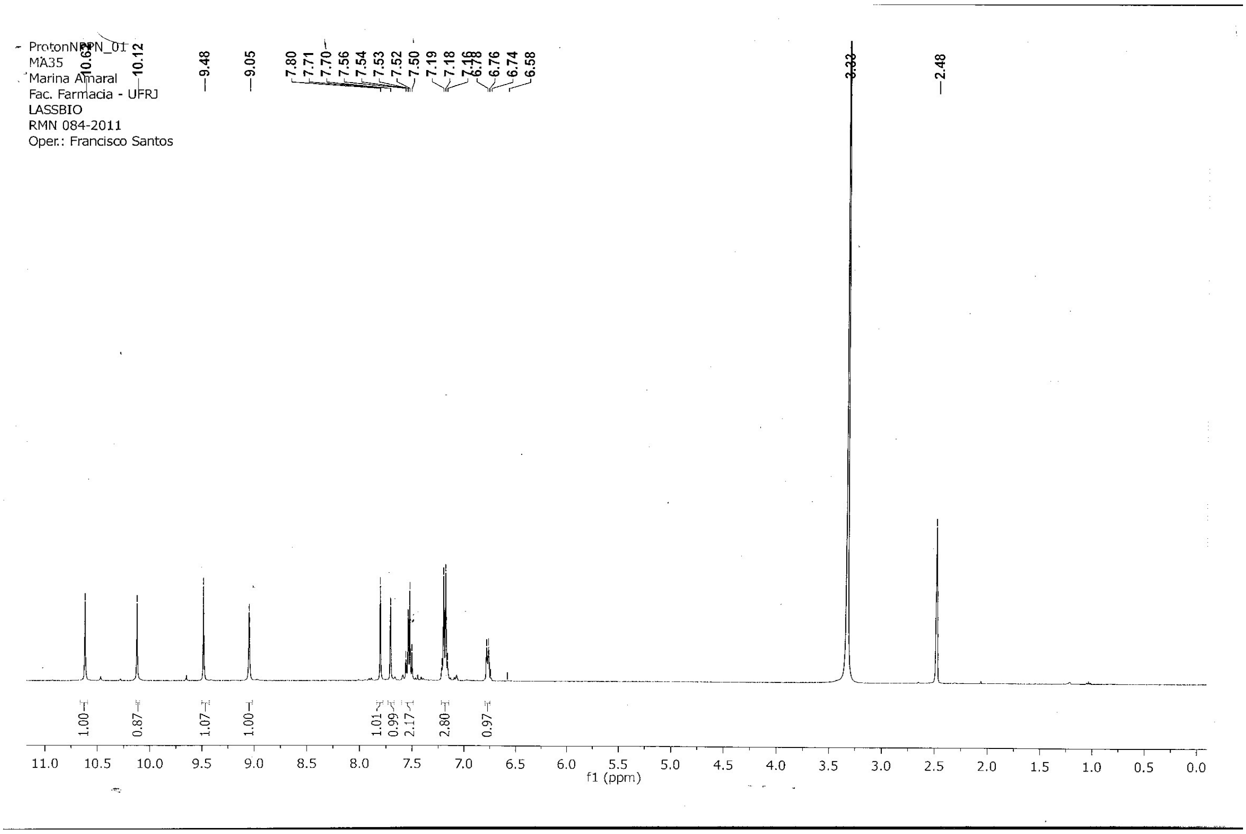

Figure S28. Compound 10c (LASSBio-1708) ^{1}H NMR

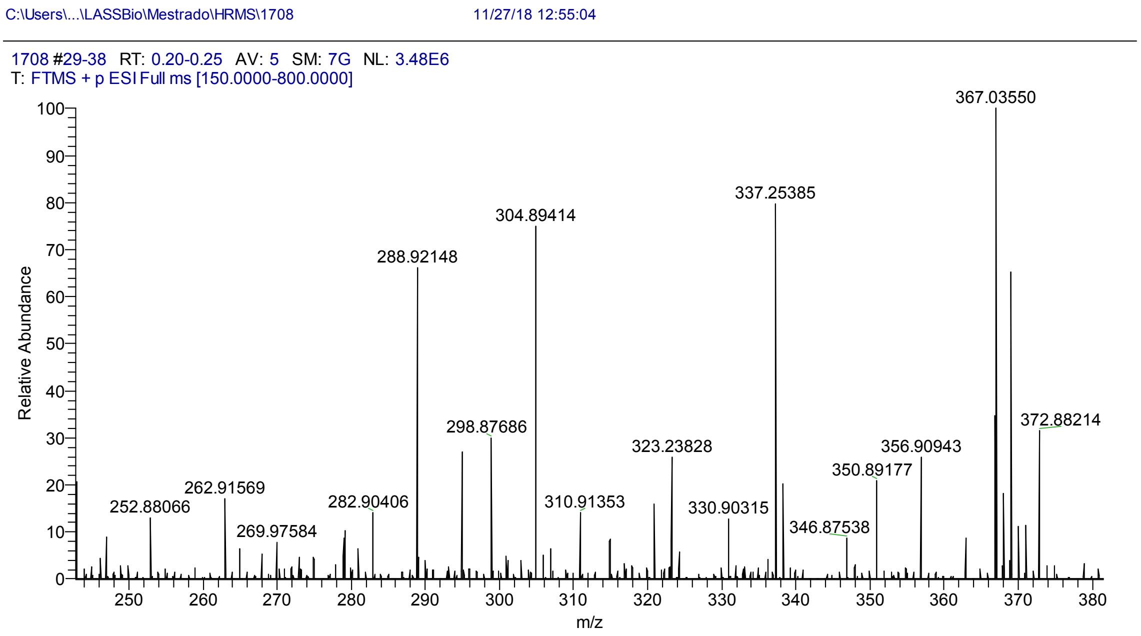

Figure S29. Compound 10c (LASSBio-1708) HRMS

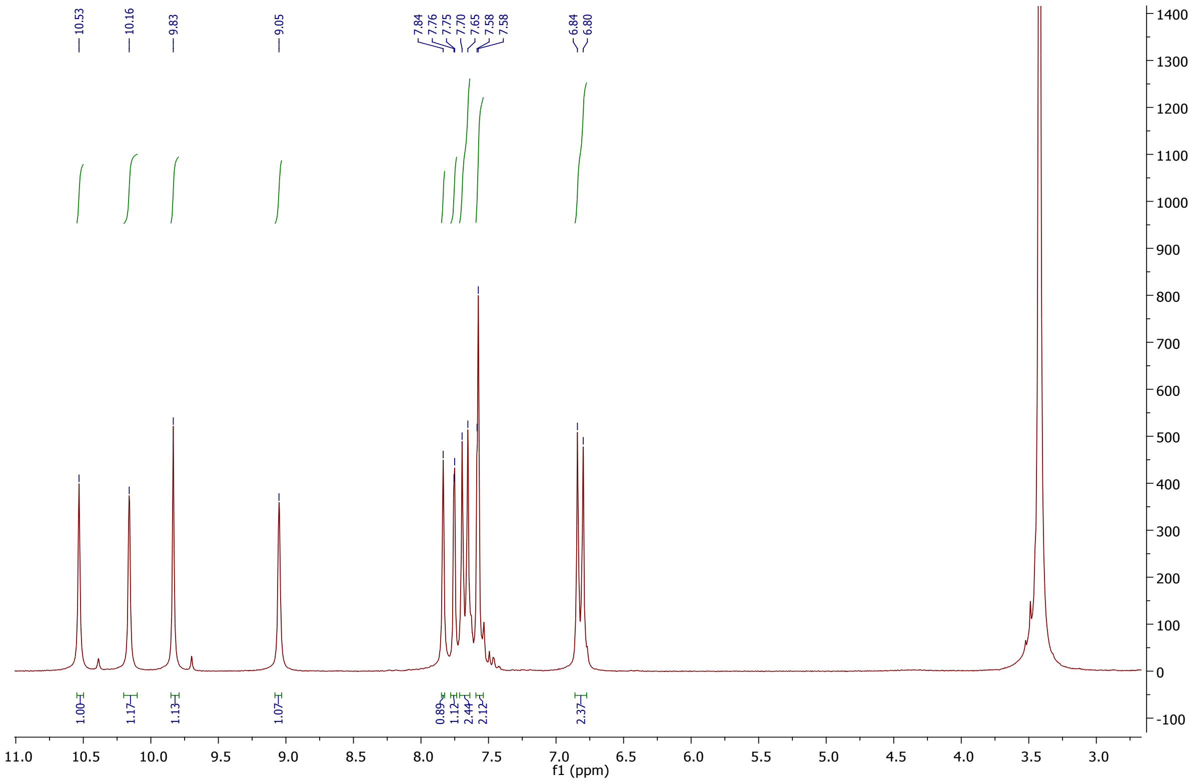

Figure S30. Compound 10d (LASSBio-1707) ^{1}H NMR

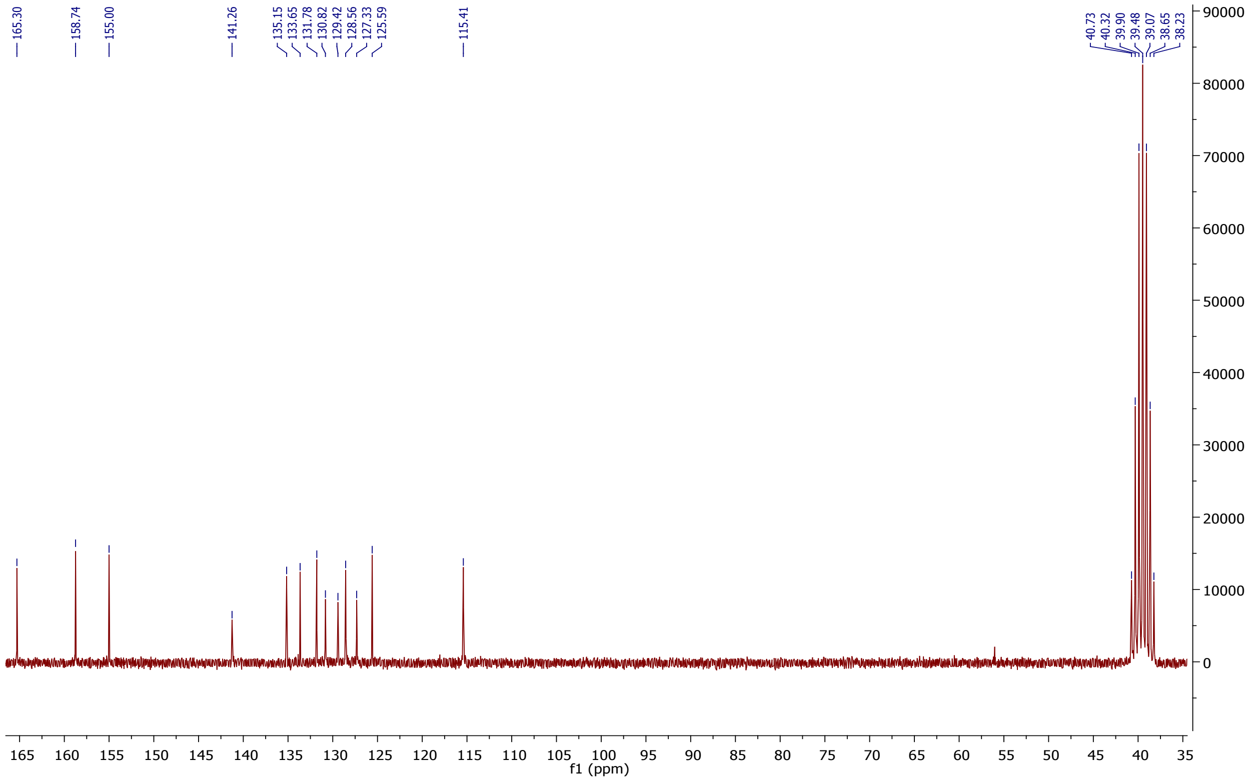

Figure S31. Compound 10d (LASSBio-1707) ^{13}C NMR

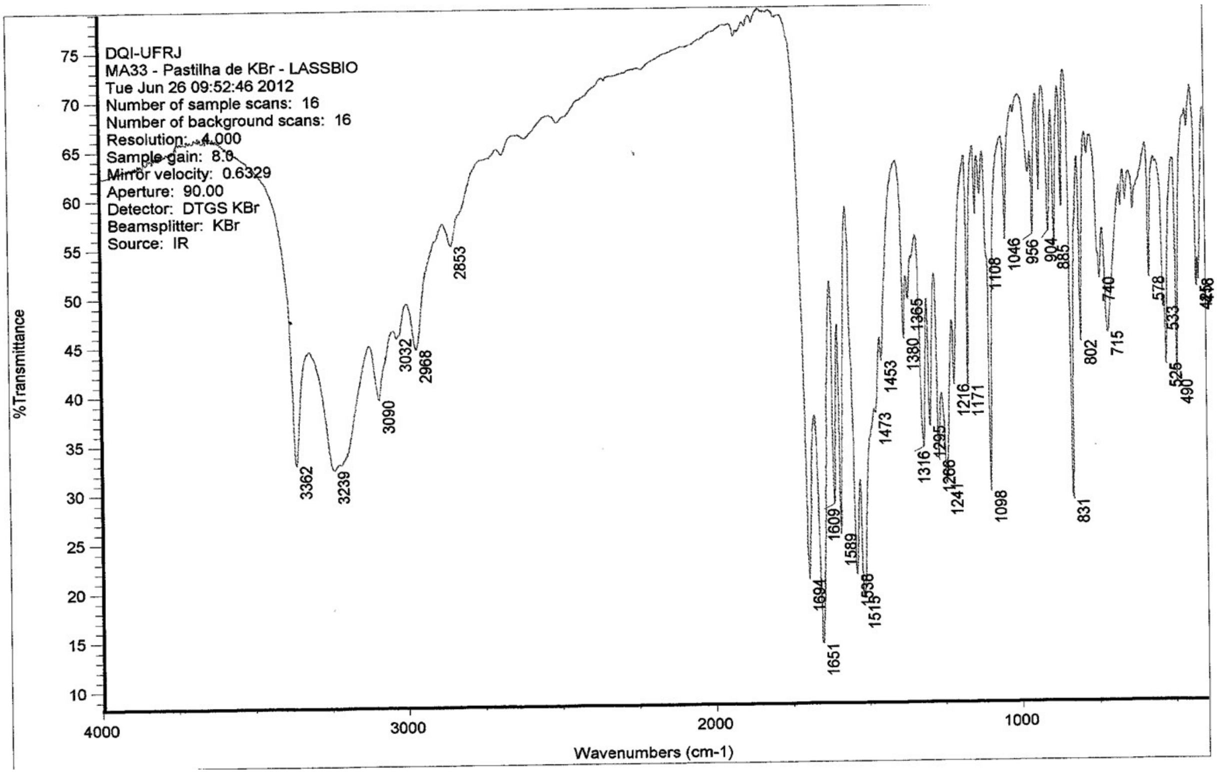

Figure S32. Compound 10d (LASSBio-1707) IR

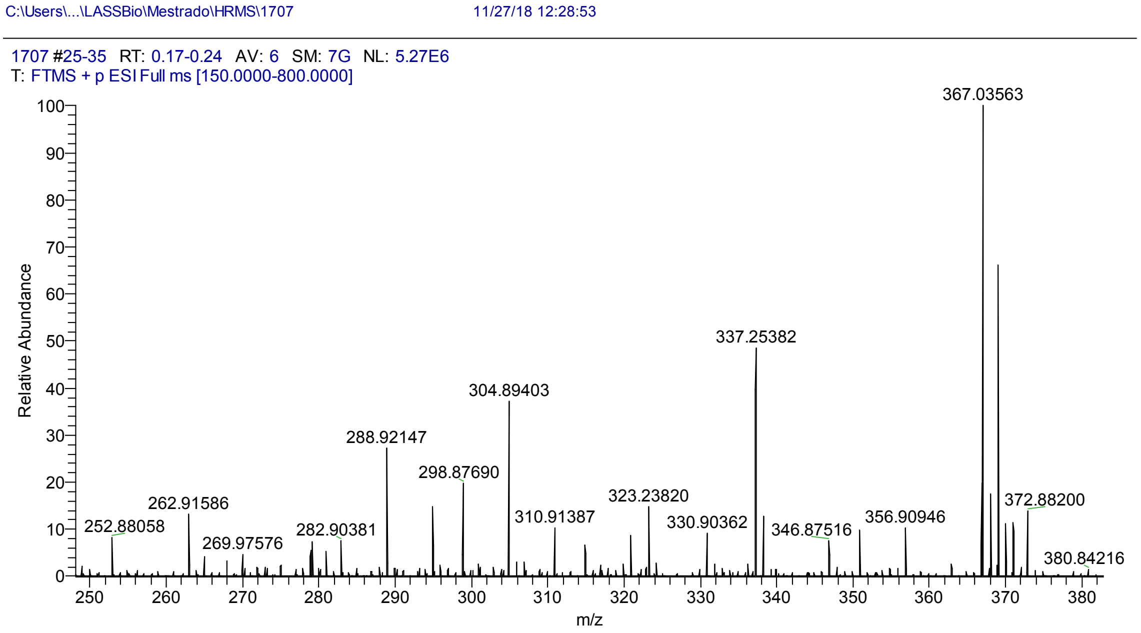

Figure S33. Compound 10d (LASSBio-1707) HRMS

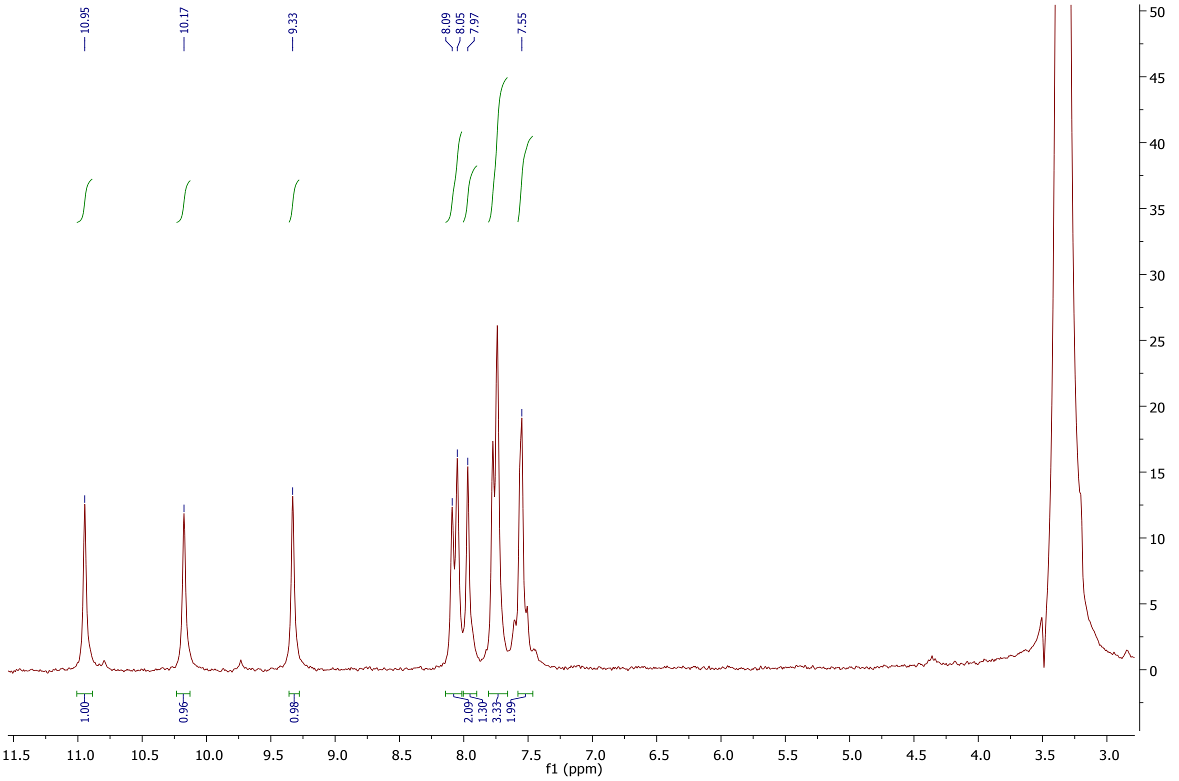

Figure S34. Compound 10e (LASSBio-1736) ^{1}H NMR

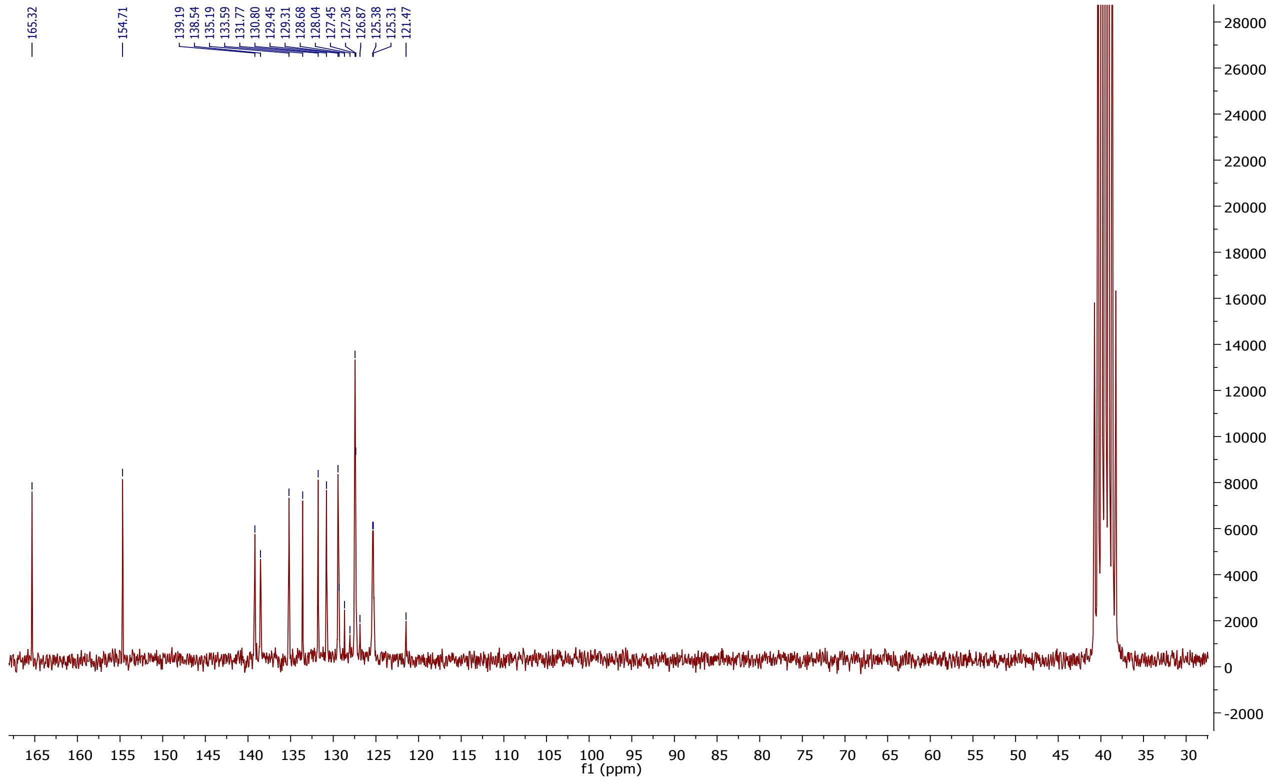

Figure S35. Compound 10e (LASSBio-1736) ^{13}C NMR

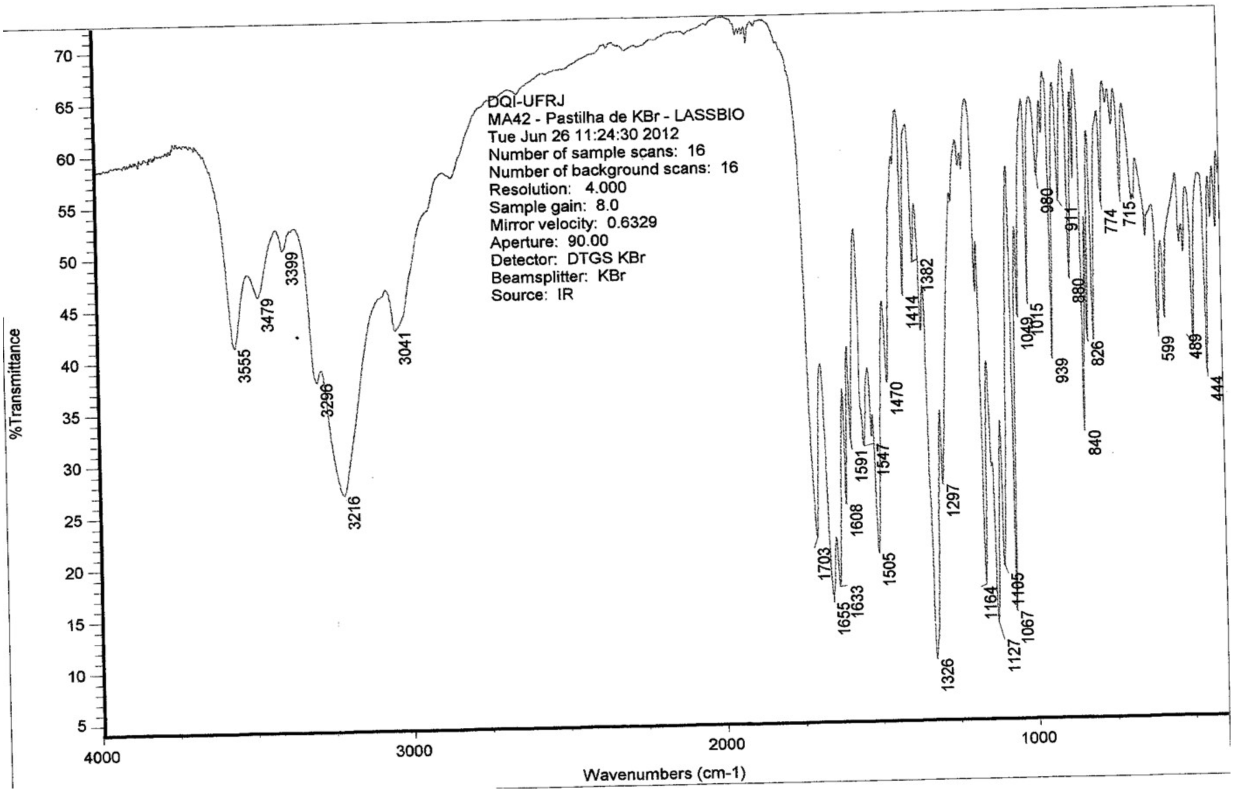

Figure S36. Compound 10e (LASSBio-1736) IR

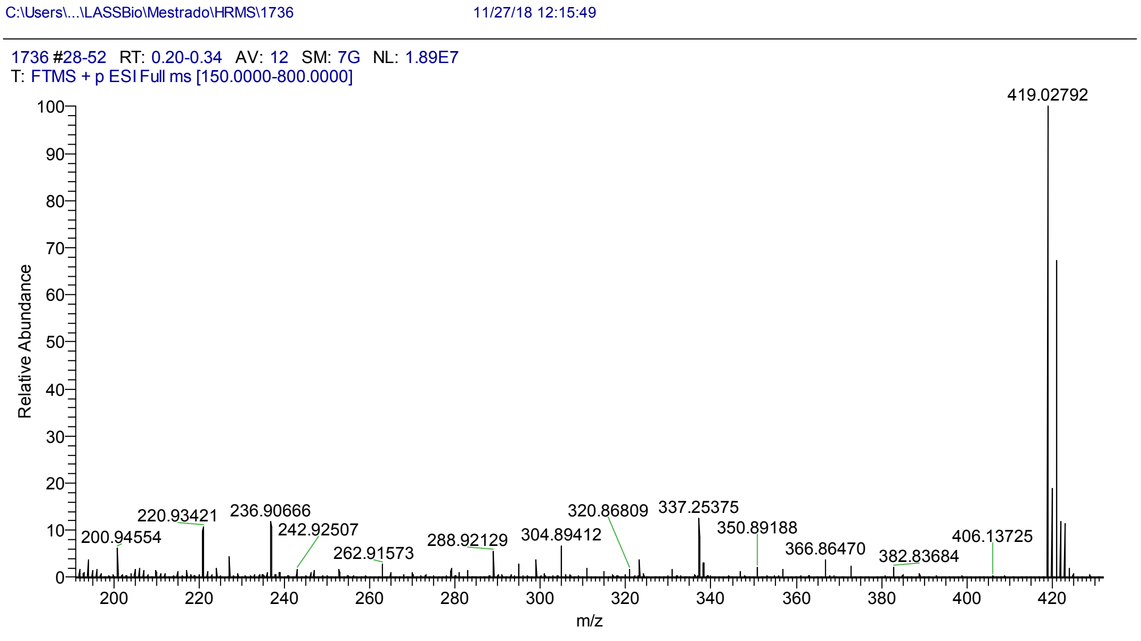

Figure S37. Compound 10e (LASSBio-1736) HRMS

Table S1. Crystal data and refinement parameters of compound 9a (LASSBio-1491).

structural formula		$(C_{13}H_{10}ClN_5O_5)_2(H_2O)$
fw (g/mol)		721.43
cryst syst		monoclinic
space group		$P2_1/c$
Z / Z'		4/2
T (K)		296(2)
unit cell dimensions	*a* (Å)	11.666(5)
	b (Å)	8.002(5)
	c (Å)	33.179(5)
	β (°)	99.513(5)
V (Å^3)		3055(2)
calculated density (Mg/m^3)		1.569
absorption coefficient μ (mm^{-1})		0.291
θ range for data collection (°)		3.101 to 26.154
index ranges	*h*	-13 to 13
	k	-8 to 9
	l	-39 to 39
data collected		35580
unique reflections		5317
unique reflections with $I > 2\sigma(I)$		2282
symmetry factor (R_{int})		0.1930
completeness to θ_{max} (%)		97.3
F (000)		1480
parameters refined		468
goodness-of-fit on F^2		0.952

final $R1$ factor for $I > 2\sigma(I)$	0.0594
final $wR2$ factor for all data	0.1689
largest diff. peak / hole (e/Å^3)	0.220 and -0.256
CCDC deposit number	1824217

Chemical stability pH 7.4

In a 10 mL tube, 5µL (250 ng/mL) of LASSBio-1736 (10e) (stock concentration = 10000 ng/mL solubilized in DMSO) was added in 195µL of PBS (pH=7.4), and the mixture was placed in a shaker at 37ºC under vigorous stirring for 0 and 240 minutes. After each reaction time, the mixture was submitted to a liquid-liquid extraction method with 800 µL TBME (tert-Butyl methyl ether)/ ethyl acetate (1:1) containing 100 ng/mL of ketoconazole (internal standard) which were then vortexed for 1 minute. The samples extracted were then centrifuged at 3000 rpm for 5 minutes and transferred 500 µL of the organic phases to clean microtubes and evaporated under a nitrogen stream at 40 °C. The samples were resuspended with 100 µL of a methanol:water mixture (7:3) containing and were placed in 2 mL vials, and analyzed by UPLC-MS.

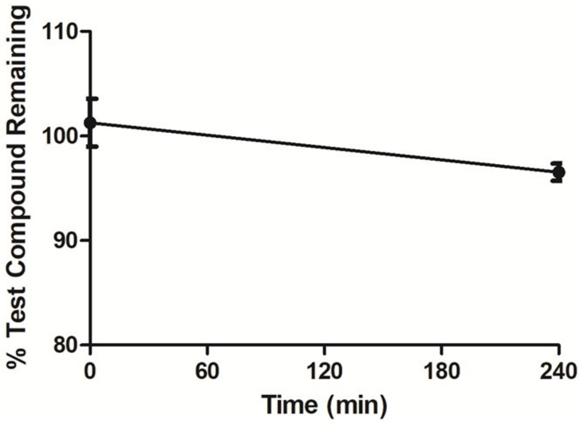

Figure S38. Chemical stability of 10e (LASSBio-1736). Data were analyzed by UPLC-MS and obtained through the compound recovery percentage expressed as mean ± standard error average of triplicates of a representative experiment.
